# A feminist critical discourse analysis of gender norms on Chinese social media: Empirical insights from WeChat public accounts

**DOI:** 10.1371/journal.pone.0338967

**Published:** 2026-01-28

**Authors:** Qingqing Tang, Xin Zhang, Taixuan Liu

**Affiliations:** 1 Department of Communication, University Putra Malaysia, Serdang, Selangor, Malaysia; 2 Department of Architecture and Landscape Design, Shandong University of Art&Design, Jinan, Shandong, China; Xi'an University of Posts and Telecommunications, CHINA

## Abstract

This study examines how gender norms are expressed and negotiated in WeChat Public Accounts using Feminist Critical Discourse Analysis (FCDA). We built a sampling frame of the ten most active gender-related accounts (June–December 2023) and screened 359 posts for relevance and analyzability. Twenty-two articles were selected for close reading. Two researchers independently coded clause- and sentence-level segments and reached agreement through discussion. Coding followed six dimensions used throughout the paper: lexical choice, modality, intertextuality, voice positioning, affective tone, and strategic silence. Four recurring themes were identified. (1) Maternal discourse and gendered discipline: texts often link women’s value to motherhood and domestic duties, combining moral language with advice on correct behavior. (2) The body and mechanisms of shame: discussions of menstruation and sexuality frequently use medical or corrective language that assigns responsibility to individual women. (3) Gender identity in cultural, legal, and policy narratives: educational, media, and policy-adjacent texts describe ideal feminine roles through procedure, quantification, and role models, which stabilize familiar expectations. (4) Resistant discourses and incremental change: some pieces re-label practices, shift speaking positions, or use humor to push back against these norms. Overall, the analysis shows how everyday textual choices can normalize gendered expectations, while limited forms of resistance also appear. The findings are bounded by the small, purposive sample and the focus on text rather than audiences or algorithms. The study provides a transparent description of patterns observed in the 22 articles and clarifies where and how resistance is articulated within them.

## Introduction

The global expansion of digital media is reshaping how gender issues are communicated and socially contested. Compared with the regional and hierarchical constraints of traditional media, digital platforms can accelerate information circulation and reorganize public engagement with gender discourses [1]. In recent years, gender perspectives have been incorporated into digital governance agendas, including in discussions by international institutions such as the United Nations, making gender an important component of evolving governance frameworks [2]. At the same time, digital media have provided marginalized communities with access to counter-publics, where survivors of gender-based violence may transcend geographic and identity boundaries to speak collectively, offer mutual support, and challenge entrenched gender myths [3]. LGBTQ + , non-binary, and transgender issues have likewise gained visibility and opportunities for recognition through digital spaces [4]. In this regard, digital media function not only as tools for communication but also as arenas in which policy negotiations, structural power, and cultural identity work intersect—with effects that vary across platforms and jurisdictions.

To situate this study within this broader landscape, it is important to clarify its purpose and significance at the outset. This article conducts a fine-grained, clause- and sentence-level analysis of how gender norms are constructed, stabilized, and challenged in texts published by WeChat Public Accounts. WeChat is analytically significant for feminist research because it constitutes one of China’s most institutionally embedded, editorially curated, and governance-sensitive communication environments, where gender discourse is shaped through a combination of platform rules, account-level curation, and affect-driven narrative practices [5,6]. Unlike open, comment-driven platforms such as Weibo or Bilibili, WeChat Public Accounts produce structured and authored long-form texts that make visible the linguistic operations—modality, stance-taking, evaluation, and strategic silence—that FCDA is uniquely suited to examine [7,8]. Examining WeChat therefore enables a deeper understanding of how feminist and anti-feminist meanings are negotiated within China’s regulated digital sphere.

Within this broader context, China’s large and fast-evolving digital environment has produced complex dynamics in gender culture. With one of the world’s largest online populations, the rapid growth of the digital public sphere has influenced gender norms and practices in multiple directions [9]. Operating within a hybrid configuration of public policy, commercial incentives, and professional norms, gender discourse production in China often reflects tensions and negotiations rather than uniform effects [6]. Meanwhile, the popularization of digital technologies has diversified gender socialization processes: interactions among educational systems, family structures, and social technologies contribute both to the continued circulation of gender stereotypes and to experiments with alternative role models [10]. Female users and activists adopt flexible strategies of expression, navigating between globalized repertoires and domestic ideological expectations [11]. More broadly, the digital sphere has reconfigured boundaries between private and public life and provides new frameworks for analyzing gender inequality in contemporary China [12]. These developments suggest that digital technologies participate in the reorganization of gendered cultural power and merit close analysis within platform-specific discursive contexts.

As a widely used component of China’s digital public sphere, the WeChat Public Accounts system plays a notable role in shaping and disseminating gender-related discourse. Through content selection, discursive framing, and affective narration, many accounts cultivate particular gendered subjectivities [13]. Under the combined influence of policy orientation and market logics, these accounts can both reproduce and discipline prevailing gender roles. Prior studies document, for instance, that discourses around “pregnancy” and “postpartum” sometimes display postfeminist sensibilities: idealized portrayals of celebrity mothers may reaffirm conventional expectations while simultaneously promoting individualized notions of independence—configurations that can align with fertility-oriented goals in specific contexts [14]. Likewise, work on popular female KOLs such as Mimeng traces neoliberal feminist rhetoric that appears to subvert male authority while also channeling women’s emotional expectations and romantic subjectivities through consumerist scripts [15]. In addition, scholarship has noted that the intersection of platform capitalism and techno-nationalism provides avenues for state actors and institutional voices to guide narratives, shaping normative femininity and acceptable gender expressions [6]. Rather than treating WeChat Public Accounts as either “open” or “enclosed,” this study approaches them as curated, subscription-driven, and account-governed channels in which regulatory signals, distributional logics, market incentives, professional norms, and formal rules operate in combination.

Globally, the gendered dynamics of digital discourse have attracted extensive scholarly attention, with critical discourse analysis (CDA) widely used to investigate gender politics on social media [16,17,18,19]. Research on China spans multiple platforms—including Weibo, Zhihu, Bilibili, and WeChat—and examines ideologies, representations, and interactional mechanisms [20,21,22,23,24].

Against this backdrop, there remains a notable gap in the existing scholarship. While prior studies have analyzed gendered representations on WeChat—ranging from postfeminist motherhood narratives [14] to neoliberal feminist rhetoric disseminated by celebrity influencers [15], and from state-guided gender norms [6] to the affective labor of female editors [25]—most work focuses on thematic or ideological patterns rather than on the micro-linguistic mechanisms through which gendered power relations are enacted. Scholarship in feminist media studies has repeatedly emphasized that linguistic choices such as modality, stance-taking, evaluation, and strategic silence play a central role in naturalizing or contesting gender hierarchies [7,8,26]. Yet these mechanisms remain underexamined in the context of WeChat’s curated, semi-closed, and institutionally embedded communication ecology.

This study addresses this gap by offering a fine-grained, clause- and sentence-level Feminist Critical Discourse Analysis of gender-related texts on WeChat Public Accounts. By foregrounding textual strategies such as lexical evaluation, intertextual positioning, affective cues, and voice configuration, the analysis seeks to illuminate how gender norms are constructed, stabilized, or resisted within China’s broader discursive contestations surrounding family roles, sexuality, demographic pressures, and feminist activism. Such platform-specific, text-level inquiry is significant not only for understanding gender governance in China but also for advancing FCDA scholarship in highly regulated digital environments [5,27]. The study addresses the following research questions:

1. What thematic patterns characterize gender discourse on WeChat Public Accounts, and how are these themes reproduced and reinforced through everyday language and narrative forms?
2. How do cultural discourses within WeChat construct specific gender identities and normative roles, and how are these identities sustained through discursive practice?
3. To what extent do resistant discourses emerge within the platform, and what are their discursive forms, linguistic strategies, and limitations in challenging mainstream gender ideologies?

## Literature review

### Gender, discourse, and social media in the context of digital media

Digital media have reshaped how gender discourses are produced, circulated, and negotiated. Social platforms operate as important arenas for role construction, identity work, and ideological contestation, while also mediating conflict and solidarity. Rather than treating “social media” as an isolated category, this study situates it within a wider digital media ecology. Compared with legacy media, many platforms can enable more flexible forms of emotional mobilization, cultural production, and visibility work, thereby contributing to emergent “digital public spheres” [28]. These spaces host individual narratives of trauma and facilitate supportive communities that share emotions, build resonance, and may foster collective reflection on cultures of gender-based violence [29].

Platformed communication, however, rarely moves in a single direction. Visual storytelling, hashtags, and affective engagement sometimes articulate gender-equality frames aligned with global governance idioms [30], while, in other cases, platforms reproduce traditional roles, including renewed patriarchal expectations, gendered divisions of labor, and recurring pushback against women’s rights claims [31,32,33]. Recent scholarship therefore attends closely to mechanisms of visibility—how recommendation systems, governance rules, and professional norms, in combination, shape what is more likely to be seen and marked as legitimate or deviant. Gaps remain in non-English contexts, in coverage of marginalized gendered identities, and in fine-grained accounts of how sentence- and clause-level language practices link up with platform mechanisms to normalize particular gender ideologies.

In China, research has shifted from protest-centered accounts to closer analyses of platform structures, cultural repertoires, and discourse mechanisms. Different platforms channel gender expression in different ways: work on Weibo documents digital feminist discourse and affective mobilization [21,34]; studies of Xiaohongshu foreground individualized and consumption-oriented logics; analyses of Bilibili often emphasize entertainment and professionalization [35]. Users also develop counter-discursive techniques through topic tags and screenshot practices under evolving governance conditions [27]. Parallel strands examine emergent gender identities and masculinities [23,36], and trace the diffusion of postfeminist narratives into mainstream spaces [15,37]. While these literatures map the broader terrain, there is still relatively limited attention to the micro-mechanics of language through which gendered norms are stabilized or unsettled.

### Gendered discourses on WeChat public accounts

Within this landscape, WeChat Public Accounts function as curated, subscription-driven, and account-governed channels that combine institutional communication with affective storytelling. Compared with more open-ended venues such as Weibo or Xiaohongshu, discourse on WeChat Public Accounts is shaped to a greater extent by editorial selection, account-level governance, and distributional rules; yet the environment is not simply closed. Gendered expressions are negotiated among everyday narration, emotional mobilization, policy signaling, market incentives, professional norms, and formal rules. This configuration makes WeChat Public Accounts a productive site for examining how gendered disciplining and ideological operations are enacted in practice.

Prior studies note that some accounts identified as feminist can, at times, reproduce consumerist frames and male-centered gazes even as they advocate independence and self-realization, thereby stabilizing familiar images of femininity [38,39,6]. In affect-driven narratives, individualized choice and responsibility are often foregrounded, aligning participation scripts with traffic-oriented engagement [15]. Editorial labor—frequently undertaken by women—has also been described as increasingly emotionalized in the pursuit of “resonance,” becoming normalized under the joint pressures of platform capitalism and gendered cultural expectations [25].

Governance is commonly characterized as a mixture of soft strategies and formal moderation. Policy cues, professional norms, and distributional nudges interact with rules-based enforcement to influence visibility [40]. In this setting, WeChat Public Accounts sometimes juxtapose figures such as the “celebrity mother” and the “independent woman,” producing postfeminist storylines that can echo pronatalist orientations while remaining compatible with platform compliance [14,39]. In discussing these dynamics, this study uses “state-led birth and development policies” to refer to policy agendas around fertility, population, and family development; in the Chinese context, such agendas often travel alongside what is sometimes described as “state feminism”—that is, a state-endorsed equality discourse that links women’s development to broader social and economic goals. The point here is not a single source of control but a combined effect: institutional voices, platform architectures, and commercial incentives jointly shape discursive visibility [6].

At the same time, WeChat Public Accounts work as spaces of women’s community-building and cultural capital negotiation. Empowerment narratives circulate alongside more subtle prescriptions for conformity with dominant gender orders and neoliberal work ethics, establishing visible models of diligence, self-discipline, emotional composure, and “balance” as markers of the “qualified woman” [41,42]. Participants often navigate tensions among different female publics, as producers of discourse and potential targets of critique, in a field where visibility can bring both recognition and backlash.

Overall, research on WeChat Public Accounts spans these strands but still pays comparatively limited attention to how language practices—at the level of grammar, metaphor, evaluation, affect, and interaction—operate in tandem with platform mechanisms to produce legitimacy and audibility. This study therefore turns to a text-level analysis of sampled posts to trace how visibility and normality are organized in the routine choices of discourse.

### Feminist critical discourse analysis

Feminist Critical Discourse Analysis (FCDA) extends critical discourse approaches by centering how language, power, and gender ideology are co-produced in routine texts and interactions [7,8]. Whereas much CDA foregrounds macro-structures of inequality, FCDA insists on the micro-operations through which gendered hierarchies are made to appear reasonable, familiar, or inevitable—through choices of transitivity and modality, patterns of evaluation and stance-taking, metaphor and affect, intertextual citation, and strategic silence. In platformed communication—marked by emotionalized storytelling, postfeminist idioms, and algorithmic curation—such textual operations are tightly entangled with distributional and compliance environments, making FCDA a precise instrument for analyzing how discursive normality is produced [43].

FCDA’s analytic pay-off is twofold. First, it traces normalization: the subtle ways grammatical patterning, narrative templates, and expertise claims sediment expectations about gendered behavior. Second, it renders counter-voices visible by following the textual devices through which marginalized subjects contest dominant frames. Studies across diverse settings demonstrate this dual focus. Revelles-Benavente [26] shows how #BringBackOurGirls politicized the semantics of “girl” and “agency,” relocating local struggles within transnational governance discourse. Boling [44] identifies how emotional language and visual storytelling on Instagram co-constitute resonance networks around #ShePersisted. Tohari [45] combines FCDA with Foucauldian perspectives to show how feminist users build counter-hegemonic spaces following patriarchal flashpoints, while Lane [46] analyzes “strategic femininity” as a pragmatic accommodation to regulatory discourses on Facebook. In non-Western contexts, Ringer [18] documents how Brazilian sex workers on Twitter negotiate identity and value amid platform precarity; in Asia, studies track how hashtagging, screenshot curation, and irony sustain micro-resistance communities [27], how influencer–audience negotiations are bounded by platform acceptability [47], and how female politicians encounter routinized harassment repertoires despite superficial inclusivity [48]. Together, this work situates FCDA as a method able to connect textual minutiae to institutional conditions.

These concerns are directly relevant to WeChat Public Accounts, an editorially curated, subscription-based, rule-governed environment embedded in institutional media logics. Unlike more open, broadcast-oriented venues, Public Accounts often cultivate engagement through affective narration and resonance-building tactics[49]. Such formats lend themselves to soft expression: seemingly moral or empowerment-oriented content that travels smoothly under algorithmic and compliance pressures while calibrating what counts as appropriate femininity or responsible choice [14]. In this setting, individualized agency talk—self-optimization, choice, emotional self-management—can align with consumer participation scripts [15], and the normalized affective labor of women-majority editorial teams may function as both production practice and gendered expectation [41]. FCDA does not presume uniform effects; rather, it enables clause-level reconstruction of how such expectations are linguistically assembled and made credible.

Methodologically, the present study operationalizes FCDA as a close reading at the sentence and clause level mapped to recurrent strategies across the corpus: (i) modality and evaluation to track how obligation, permission, and moral worth are allocated; (ii) voice and perspective to distinguish authorial assertion from quoted expertise and aggregated authority; (iii) intertextuality and citation to identify policy, medical, and celebrity resources that legitimate claims; (iv) metaphor, affect, and silence to capture how care, risk, shame, and pride are mobilized—or backgrounded—in ways that organize consent. Interpreting these textual choices alongside account-level governance and distributional cues clarifies how particular gendered positions become sayable, visible, and legitimate on the platform.

## Methodology

### Data sources and sampling strategy

This study employed Newrank.cn to identify gender-related long-form posts published by WeChat Public Accounts. Newrank is widely used in communication research as a third-party analytics tool capable of mapping platform activity and capturing readership indicators [50]. Consistent with qualitative inquiry, the purpose of using Newrank was not to generate frequency estimates but to locate authored, discursively rich texts suitable for FCDA.

The sampling window (June–December 2023) was bounded for interpretive rather than statistical reasons. A six-month period generated a sufficiently coherent body of long-form posts that allowed for clause-level reading while avoiding cross-year policy shifts that may introduce interpretive noise. Treating the corpus as an analytically bounded snapshot aligns with FCDA’s emphasis on contextual depth rather than prevalence.

Table 1 lists the ten accounts monitored to map the discursive ecology during the sampling window. These accounts serve as the sampling frame, not the final sample. From the 359 retrieved items, the study applied FCDA-oriented screening criteria: posts were excluded when they consisted of brief announcements, event summaries, or highly fragmented texts lacking authorial narrative continuity.

**Table 1:** The top 10 WeChat public accounts.

WeChat public account	Article number
Gender studies horizon	1-87
Gender and society studies	88-103
Gender dream	104-196
Cultural resistances	197-203
A green apricot	204-237
Common language	238-242
Sencha group	243-256
His voice	257-340
Gender and law program	341-343
Lingnan queer	344-359

**Fig 1:**
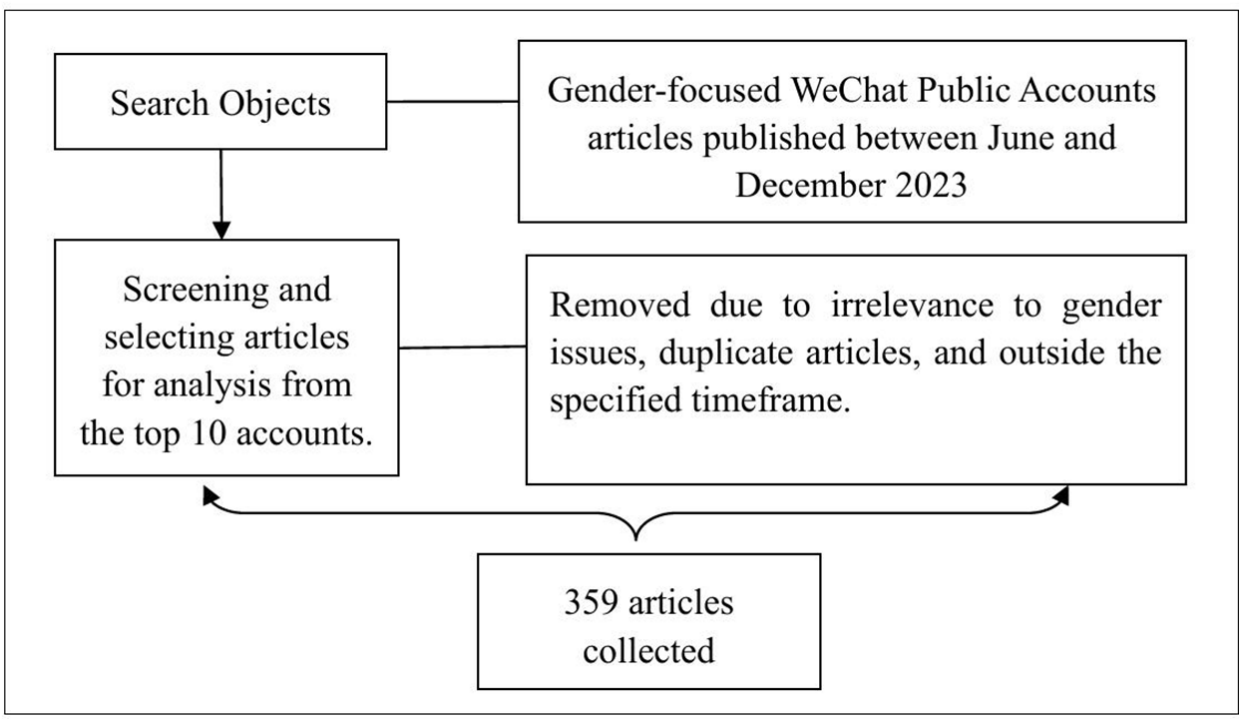
Flow diagram of the search strategy.

From this frame (359 items), the study screened posts for close reading. Items were excluded when they were brief notices, event digests, or lacked continuous authorial prose suitable for sentence-level interpretation. Fig 1 illustrates the article retrieval strategy flowchart.

Building on this procedure, the study selected 22 articles for detailed FCDA. Table 2 documents the final analytical corpus and corresponding source accounts. No quotas were imposed by account identity or readership size, as FCDA privileges discursive density and ideological salience over distributional representativeness.

**Table 2:** Selected articles for feminist content discourse analysis.

No.	WeChat public account	Article title
1	Gender studies horizon	Gender mainstreaming: a strategic approach to safeguarding women’s rights in the digital era
2	A green apricot	Not wanting a daughter—is that misogyny?
3	A green apricot	After three months of struggle, I finally cut my hair Short
4	Gender studies horizon	What factors should be considered when returning the bride price?
5	Gender studies horizon	Supreme court and all-China women’s federation release model cases of domestic violence crimes
6	Gender studies horizon	“My dad is a man”: the pitfalls of gender education
7	Gender studies horizon	Unlocking the power of gender equality to create infinite possibilities
8	A green apricot	Can we move beyond the supremacy of sexual attraction?
9	A green apricot	After a year of gender-free dating, I turned my boyfriend into a “Girlfriend”
10	Gender studies horizon	New trends and characteristics in China’s marriage and family structures
11	A green apricot	Reflections on physical competition shows: do women need to be strong to earn respect?
12	Gender studies horizon	Changing patterns of childbearing intentions among Chinese women of reproductive age
13	Gender dream	After menstruation “left,” Xiao fu discovered a new way of relating to it
14	Gender dream	How can asexual perspectives inform our understanding of sex education?
15	Gender dream	Expectation, limitation, and identification: why do children align with traditional gender roles?
16	A green apricot	Watching a childhood video of myself running naked, I felt deep shame...
17	Gender studies horizon	The internet as a new frontier for women’s development
18	Gender studies horizon	2023 Forum on women pioneers in science and technology innovation
19	Gender and society studies	Why do highly educated women become full-time mothers?
20	Gender and society studies	Constructing the new mother: The formation and practice of child-rearing knowledge in modern China (1900–1937)
21	Gender and society studies	“Leftover women” in matchmaking corners: caught between tradition and modernity
22	Gender and society studies	Revealing hidden labor

**Fig 2:**
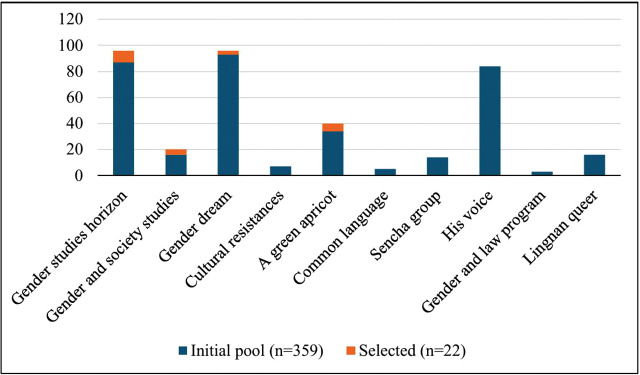
Initial sampling frame (n = 359) and analyzed subset (n = 22) by account*. *Totals per account are calculated from the article-ID ranges in Table 1; inclusions are counted from Table 2. The figure clarifies the linkage between the frame and the analyzed sample and explains why some accounts have no eligible items after applying analytic-suitability criteria.

Fig 2 further illustrates how the analyzed subset relates to the retrieval frame: some accounts yielded no texts not because of exclusion but because all retrieved items lacked the narrative continuity required for sentence-level analysis.

To enhance transparency and assess the representativeness of the purposive sample, the study report basic exposure indicators—*total reads, likes, forwards, and comments*—for each of the 22 articles (Table 3). These values are drawn from the platform counters at the time of capture and provide a proxy for audience reach and engagement. Selection did not favor high-readership items; instead, it prioritized discursive analyzability. They situate the qualitative FCDA analysis in relation to circulation patterns on WeChat Public Accounts.

**Table 3:** Exposure metrics of the FCDA sample articles.

Article title	Reads total^a^	Likes total^a^	Forward total^b^	Comments
Gender mainstreaming: a strategic approach to safeguarding women’s rights in the digital era	302	8	13	0
Not wanting a daughter—is that misogyny?	10000	91	4	36
After three months of struggle, I finally cut my hair Short	7683	43	1	20
What factors should be considered when returning the bride price?	144	0	21	0
Supreme court and all-China women’s federation release model cases of domestic violence crimes	382	10	59	0
New Occupations and Business Models Drive New Developments in Women’s Employment	347	2	31	0
Unlocking the power of gender equality to create infinite possibilities	375	3	2	0
Can we move beyond the supremacy of sexual attraction?	12000	23	66	7
After a year of gender-free dating, I turned my boyfriend into a “Girlfriend”	13000	172	5	8
New trends and characteristics in China’s marriage and family structures	551	1	24	0
Reflections on physical competition shows: do women need to be strong to earn respect?	5050	40	44	4
Changing patterns of childbearing intentions among Chinese women of reproductive age	992	5	37	0
After menstruation “left,” Xiao fu discovered a new way of relating to it	700	12	3	Not open
How can asexual perspectives inform our understanding of sex education?	181	8	9	Not open
Expectation, limitation, and identification: why do children align with traditional gender roles?	321	14	32	Not open
Watching a childhood video of myself running naked, I felt deep shame...	7058	25	2	11
The internet as a new frontier for women’s development	288	43	5	0
2023 Forum on women pioneers in science and technology innovation	330	7	26	0
Why do highly educated women become full-time mothers?	1634	34	18	0
Constructing the new mother: The formation and practice of child-rearing knowledge in modern China (1900–1937)	853	5	17	0
“Leftover women” in matchmaking corners: caught between tradition and modernity	2268	38	28	1
Revealing hidden labor	1659	45	26	0

^a^Reads Total and Likes Total are platform counters as captured during the study window.

^b^Forward Total refers to the number of shares displayed on the platform at the time of capture.

### Reliability, reflexivity, and interpretive rigor

FCDA is interpretive and reflexive by design. Accordingly, the study adopted practices that foreground researcher positionality and interpretive depth rather than procedural standardization alone. As women scholars trained in gender studies and discourse analysis, the research team acknowledged that our readings of gendered language—especially moralizing, bodily regulation, or maternal discourses—are shaped by our social locations and prior engagements with feminist scholarship. To make this interpretive labor visible, reflexive memos were written during coding to document how particular assumptions, reactions, or uncertainties shaped emergent interpretations.

To enhance interpretive coherence, two researchers independently coded an initial subset of texts to refine the analytical categories. Rather than seeking mechanical agreement, discrepancies were examined through negotiated consensus, which served as a site of interpretive reflexivity: alternative readings were compared, tensions were recorded, and minority interpretations were preserved where analytically meaningful. These discussions strengthened awareness of textual ambiguity and prevented premature closure.

Consistent with SRQR guidelines, double coding, memo-based reflexivity, and peer debriefing were incorporated not to impose quantitative reliability but to maintain transparency regarding how interpretive claims were formed [51].

### Analytical framework and procedure

The study followed Feminist Critical Discourse Analysis [7,8], operationalized through Nartey’s (2020) three-stage procedure of text selection, segmentation, and interpretive coding. FCDA is concerned with how linguistic choices enact gendered power relations; thus, the unit of analysis was the discourse segment—sentences or short passages that articulated, normalized, or challenged gender ideologies.

Each segment was examined along six FCDA dimensions: lexical choice, modality, intertextuality, voice positioning, affective tone, and strategic silence. These dimensions were selected because they make visible the subtle operations of power—how obligation is encoded, how authority is invoked, how emotions regulate social expectations, and how silence functions as an ideological device. Table 4 summarizes the operationalization of each dimension.

**Table 4:** FCDA coding dimensions.

Dimension	Operationalization	Example cue
Lexical choice	Evaluative or stigmatizing terms	“厌女症” (misogyny), “毒瘤” (tumor)
Modality	Obligation/necessity markers	必须 (must), 应当 (should), 不得 (must not)
Intertextuality	Explicit references to laws, policies, statistics, expert discourse	联合国妇女署报告(UN Women Report)
Voice positioning	Attribution to institutional vs. individual speakers	“最高人民法院认为…” (The court held…)
Affective tone	Emotional intensification, evaluative affect	“巨大伤害”(Extremely severe), “极其严重”(Extremely serious)
Strategic silence	Expected references (agency, consent, structural conditions) absent in local context	No mention of women’s consent in marriage texts

Two researchers independently annotated all 22 articles, compared segment-level coding, and reconciled interpretive tensions through memo-supported discussion. Table 5 presents five representative coded excerpts to illustrate how FCDA principles were applied in practice. Rather than serving as mechanical categories, the six dimensions functioned as analytical lenses that guided the transition from micro-level linguistic cues to thematic interpretations presented in the Results section.

**Table 5:** Illustrative coded excerpts.

Article ID	Excerpt (Chinese → English gloss)	Applied codes	Analytical memo
1	“必须确保在技术的构想、设计、开发、部署、评价和监管过程中促进、尊重和实现人权” → *“[We] must ensure that human rights are promoted, respected, and realized in all phases of technology governance.”*	Modality; Intertextuality; Voice positioning	Institutional register mandates obligation; reference to global governance frames gender equality as a policy imperative.
2	“那些因为爱女儿, 所以不想生女儿…其实是厌女症的表现” → *“Those who avoid having daughters out of love for their daughters… are in fact displaying misogyny.”*	Lexical choice; Modality; Affective tone	The diagnostic label “厌女症” (“misogyny”) functions as a stigmatizing lexical choice; presupposed obligation that daughters will suffer gendered burdens; affective framing through fear and pity.
3	“剪成寸头后感觉轻松了很多…家人却说‘剪成男孩子喽’” → *“After cutting my hair short I felt relieved… but my family said ‘now you look like a boy.’”*	Affective tone; Lexical choice; Resistance	First-person affective narration highlights relief and agency; family’s evaluative comment polices femininity; excerpt illustrates resistance through embodied practice.
4	“审理法院认为… 彩礼蕴含祝福, 但高额彩礼背离社会价值” → *“The court held… that bride price contains blessings, but excessive amounts violate social values.”*	Voice positioning; Lexical choice; Intertextuality	Legal voice authorizes gender norms; moral adjectives (“背离/violate”) frame deviance; institutional intertextuality anchors legitimacy.
5	“人身安全保护令绝非一纸空文, 无视禁令必被严惩。” → “The personal safety protection order is by no means an empty piece of paper; defying the prohibition will inevitably be severely punished.”	Modality; Lexical choice; Voice positioning	Judicial modality enforces obligation; evaluative intensifiers (“绝非/必/严惩”) amplify deterrence; institutional voice constructs state authority against gender violence.

In subsequent sections, these coded dimensions serve as the basis for thematic analysis, linking micro-level discursive devices to broader patterns of gendered power, regulation, and resistance in the WeChat Public Account sphere.

## Results

Through an initial round of classification and thematic mapping, four dominant themes of gender discourse were identified, offering a structural overview of how gender-related issues are distributed across the sample texts (see Table 6). This thematic categorization not only provides a macro-level perspective for the subsequent analysis but also reveals both the diversity of gender discursive patterns and the concentrated focus on specific gender issues within the dataset.

**Table 6:** Gendered discourse themes in the 20 selected WeChat articles.

No.	Article title	Gendered discourse theme
1	Gender mainstreaming: a strategic approach to safeguarding women’s rights in the digital era	Gender Identity Construction in Cultural Discourse
2	Not wanting a daughter—is That misogyny?	Gender Identity Construction in Cultural Discourse
3	After three months of struggle, I finally cut my hair Short	The Body and Mechanisms of Shame
4	What factors should be considered when returning the bride price?	Gender Identity Construction in Cultural Discourse
5	Supreme court and all-China women’s federation release model cases of domestic violence crimes	Gender Identity Construction in Cultural Discourse
6	New Occupations and Business Models Drive New Developments in Women’s Employment	Resistant Discourses and Discursive Transformation
7	Unlocking the power of gender equality to create infinite possibilities	Gender Identity Construction in Cultural Discourse
8	Can we move beyond the supremacy of sexual attraction?	Resistant Discourses and Discursive Transformation
9	After a year of gender-free dating, I turned my boyfriend into a “Girlfriend”	Resistant Discourses and Discursive Transformation
10	New trends and characteristics in China’s marriage and family structures	Gender Identity Construction in Cultural Discourse
11	Reflections on physical competition shows: do women need to be strong to earn respect?	Body discipline, gender Norms, and shame discourse
12	Changing patterns of childbearing intentions among Chinese women of reproductive age	Gender Identity Construction in Cultural Discourse
13	After menstruation “left,” Xiao fu discovered a new way of relating to it	The Body and Mechanisms of Shame
14	How can asexual perspectives inform our understanding of sex education?	Gender Identity Construction in Cultural Discourse
15	Expectation, limitation, and identification: why do children align with traditional gender roles?	Gender Identity Construction in Cultural Discourse
16	Watching a childhood video of myself running naked, I felt deep shame...	The Body and Mechanisms of Shame
17	The internet as a new frontier for women’s development	Resistant Discourses and Discursive Transformation
18	2023 Forum on women pioneers in science and technology innovation	Gender Identity Construction in Cultural Discourse
19	Why do highly educated women become full-time mothers?	Maternal Discourse and Gendered Discipline
20	Constructing the new mother: The formation and practice of child-rearing knowledge in modern China (1900–1937)	Gender Identity Construction in Cultural Discourse
21	“Leftover women” in matchmaking corners: caught between tradition and modernity	Maternal Discourse and Gendered Discipline
22	Revealing hidden labor	Resistant Discourses and Discursive Transformation

The analysis in this chapter is guided by the central triad of FCDA—language, power, and ideology—and seeks to uncover how gendered ideologies are produced, reinforced, and contested through discourse. These themes also reflect key tensions in contemporary gender debate in China, including demographic pressures, shifting expectations of family roles, contested norms of bodily autonomy, and the increasingly negotiated space for feminist expression within regulated digital environments. Rather than presenting a linear categorization, the discussion proceeds through four interrelated thematic point that emerged from the corpus.

### Maternal discourse and gendered discipline

In the context of China’s ongoing public debates over fertility policy, family roles, and “population responsibility,” maternal discourse remains a central axis of gender politics. The texts in this corpus draw heavily on these broader debates, reflecting how motherhood continues to serve as a key site where responsibility, virtue, and civic contribution are discursively negotiated.

Across the corpus, motherhood frequently appears as a key site where language allocates responsibility and moral worth. Lexical choices such as “voluntary resignation,” “prioritizing family,” or being “better suited to care” individualize outcomes that may be strongly conditioned by childcare access, employer flexibility, or household resources. When paired with obligation-laden modality (“should,” “must,” “inevitable”), these terms tend to recast adjustable arrangements as personal virtue or failure, softening the visibility of institutional levers.

A second strand involves managerial metaphors that seek to revalue care—“CEO of the household,” “Director of Education,” and related labels. These moves reposition voice from dependency to competence and sometimes counter the stigma attached to staying at home. Yet the distributional horizon often remains narrow: caregiving is feminized, alternatives such as shared schedules or paternal leave are less articulated, and the imagined solution space is contained within women’s time and affect. When historical vignettes surface—e.g., scientized labels for maternal roles or nation-oriented descriptions of reproductive contribution—they elevate motherhood as public service but background spousal reciprocity. We read these episodes as rhetorical resources rather than as a uniform script; their presence and strength vary by account type.

The normative reach of maternal discourse is not limited to mothers. Accounts addressing unmarried or childfree women sometimes bind “marriageable age” to ideas of respectability, aligning timelines with moral valuation. In the same dataset, however, counter-voices reframe career achievement, deferred marriage, or alternative partnerships as legitimate trajectories. These texts remind us that subject positions are negotiated rather than fixed, and that age, occupation, and locality modulate how judgments attach.

Taken together, maternal discourse in the sample works through everyday mechanisms—lexical naturalization, the modality of inevitability, and the silence around redistributive options—to personalize structural constraints. These discursive tendencies align with China’s wider debates about fertility incentives, gendered labor division, and state-endorsed family norms, revealing how digital texts contribute to the normalization of caregiving as women’s moral and civic duty. Pockets of re-articulation do appear, but they operate within the same visibility conditions that the broader discourse helps to maintain.

### The body and mechanisms of shame

Debates around bodily autonomy, privacy, and sexual education have become increasingly visible in China’s public sphere, especially as feminist and youth communities challenge conventional moral frameworks. The corpus reflects these tensions, showing how narratives about the female body often reproduce long-standing anxieties about propriety and control.

The female body constitutes a second arena in which power often proceeds under the cover of neutrality and science. Reports on women in physically demanding competitions sometimes emphasize that rules set “no gender restrictions,” yet the implicit benchmark remains male-coded performance. Equality is then recast as assimilation: to qualify as “neutral,” women are asked to align with a standard that was not designed around their embodied experience. This effect is not asserted as universal; it appears where narration and quoted authority privilege a single referent body.

Medicalized talk about menstruation displays a related dynamic. Metaphors of unruliness or unreliability, combined with standardized prescriptions, can shift burden from institutions to individuals. Emotional and logistical costs—rest, pain management at work or school, access to products—are less elaborated than clinical protocols, producing a sense of even-handedness that downplays uneven impact across gender and class. Where doctor–patient exchanges are summarized, the professional voice frequently occupies the authorial position; experiential voice is quoted more sparingly.

Sexuality-education texts are mixed rather than uniform. Some narratives cast boys as carefree and girls as vigilant, distributing shame asymmetrically and minimizing boys’ privacy needs. Others call for balanced curricula that cultivate bodily awareness for all children. Family joking and permissiveness around boys’ bodies, when unaccompanied by guidance, tend to normalize girls’ self-monitoring as a default. These patterns are likely mediated by resources: households with limited space or time face different constraints from those with flexible arrangements.

Counter-discourses reframe menstruation as self-knowledge and boundary setting, and propose redesigns to privacy education that foreground mutual respect. Their reach is uneven, but they indicate that shame is a discursive outcome rather than an inevitability and that textual choices can recalibrate where the burden falls. These dynamics mirror broader gender debates in China regarding bodily regulation, menstrual stigma, and the limits of institutional recognition of women’s embodied experiences.

### Gender identity construction in cultural discourse

China’s contemporary gender landscape is marked by contestations over “appropriate femininity,” the role of women in national development agendas, and the increasing collision between market-driven self-making and policy-driven family expectations. The texts analyzed here reproduce these debates through cultural, legal, and educational narratives that stabilize particular gender identities.

Cultural, legal, and policy texts in the corpus often combine managerial vocabulary with warm moral registers, positioning women as responsible contributors to household stability and social development. Lexical frames such as “activating potential,” “meeting societal needs,” or “serving development” render participation sensible and necessary, while the arrangement of voices centers institutional or expert authority. Women’s voices appear, but more often as implementers than as agenda-setters, especially in programmatic genres.

Legal narration—around marriage payments, reproductive harms, or family obligations—frequently translates embodied or emotional labor into countable terms. This economization can clarify dispute parameters; it can also narrow the lens to calculable loss and gain, leaving structural inequalities less visible. We do not claim that such framing is constant; some cases foreground context and care, while others emphasize procedure and quantification.

Educational and media materials sometimes reproduce binary imagery—color-coded toys, gendered classroom examples—and provide limited space for non-heteronormative expression. Silences of this kind are not everywhere present, but where they appear they compress the range of intelligible identities. Differences by class and locality are notable: urban professional contexts foreground balance and self-optimization; rural or migrant settings more often surface concerns about cost, stigma, and service access. The result is not homogeneity but differentiated pathways through which responsibility and legitimacy are narrated.

Importantly, policy-aligned empowerment talk recurs as a further layer of normalization. Phrases like “releasing women’s potential” or “improving population quality” tie recognition to demographic and development agendas. We avoid specialist labels here; the point is that public-facing texts can enlist empowerment language to make participation feel both desirable and obligatory, with moral warmth and managerial reason working in tandem. This interplay between empowerment discourse and developmentalist logic reflects broader tensions in China’s gender debate, where recognition of women’s contributions is often articulated within state-centered or demographic frameworks rather than through claims for autonomy or structural reform.

### Resistant discourses and discursive transformation

Despite regulatory constraints and the shrinking spaces for overt feminist activism in China, digital users continue to develop subtle, tactical forms of resistance. The texts in this corpus reflect these broader tendencies, where resistance often takes negotiated, everyday, and implicit forms rather than explicit confrontation.

Resistance in the corpus is typically incremental and pragmatic rather than declarative. One pattern is lexical re-signification: familiar labels are repurposed—sometimes with irony—to expose their disciplinary edge. Another is voice repositioning, where lived experience is foregrounded as evidence against naturalized norms, especially in accounts of care, work, and bodily boundaries. Relationship talk also exhibits micro-adjustments; neutral terms such as “partner” stand in for gendered labels, and expectations are renegotiated through humor or deliberate ambiguity.

Bodily practices—short hair, clothing choices, participation in sports—are narrated as boundary setting rather than defiance, with attention to how family members and colleagues mediate acceptance. Small-business stories and craft entrepreneurship recast domestic or informal labor as economic agency, though they remain entangled with traffic incentives and compliance rules. These moves create pockets of maneuver without dissolving the conditions that circumscribe them.

Finally, the corpus gestures to uneven geographies of recognition. International celebrations of women in technology circulate widely, while Chinese experiences are less visible in those same venues. This asymmetry suggests that re-articulation travels across platforms and languages with differing traction. Even so, the texts examined here show how alternative subject positions are carved out through tone, naming, and stance—bending dominant frames rather than breaking them outright.

These forms of micro-resistance resonate with broader patterns observed in China’s digital feminist practices, where users employ irony, reframing, and strategic ambiguity to carve out space for alternative gender subjectivities within a tightly governed media environment.

## Discussion

This study used Feminist Critical Discourse Analysis to examine how gendered positions are articulated in a bounded set of WeChat Public Account texts. Rather than claiming platform-wide prevalence, the contribution is analytic and text-level: we specify how wording choices, modality and evaluation, intertextual citation, stance and voice, affective cues, and strategic silence often combine with account governance and distributional logics to render particular gendered positions sayable, visible, and legitimate.

Regarding maternal discourse, the corpus shows recurrent use of “free choice,” “self-sacrifice,” and intensified caregiving ideals. These expressions appear to acknowledge agency while tending to personalize outcomes that are also shaped by childcare access, employer flexibility, and household resources. Read alongside Bailey’s [52] observation that digital communities can recirculate the ideal of the “perfect mother,” our texts suggest a similar mechanism: moralized language narrows the solution space to individual virtue. Importantly, the strength and direction of this logic vary. Urban professional accounts foreground “balance” and self-optimization; narratives oriented to migrant or working-class settings more frequently surface cost, time scarcity, and service access. Expectations of marriage and reproduction also intersect with sexuality: non-heteronormative trajectories receive limited recognition in family and workplace scenarios, amplifying the disciplining force of “appropriate timelines.” These differences caution against treating maternal discourse as homogeneous; they indicate patterned variation across classed and sexualized life worlds.

For the body and mechanisms of shame, neutrality claims (“no gender restrictions,” “scientific management”) sometimes mask a male-coded benchmark. Equality is then recast as assimilation to a standard that does not fully accommodate menstruation, pain management, or recovery. Menstruation is framed through metaphors of unruliness while standardized prescriptions downplay uneven costs at work or school. Here, intersectional differences matter: the burden of privacy management and product affordability falls unevenly across households, and is magnified for students, shift workers, and lower-income groups. Scholarship in other platform contexts documents “de-shaming” strategies and body-positive framings (e.g., [53,54]). We reference these as indicative contrasts rather than definitive benchmarks: our concern is the mechanism by which medicalized neutrality and standardized guidance can relocate responsibility to individual bodies under specific governance conditions.

Turning to identity positioning within cultural, legal, and policy narratives, the texts frequently join managerial vocabulary with warm moral registers. Phrases such as “activating potential,” “serving social development,” or “meeting societal needs” make women’s participation feel both sensible and necessary, while voice arrangements center institutional or expert authority. Legal storytelling around marriage payments or reproductive harms can translate emotional and bodily labor into quantifiable terms, which clarifies disputes yet may bracket structural inequalities. Again, variation is visible: some accounts emphasize care and context; others foreground procedure and metrics. Policy-aligned empowerment talk is a recurring device. Rather than invoking specialist labels, we describe what the texts do: they align recognition with demographic and development agendas, tying legitimacy to household stability and civic contribution. Space for non-heteronormative identities is limited in the sample, and when present it is more often addressed as exception than norm—another site where sexuality intersects with institutional tone.

Resistant practices in the corpus are generally incremental and pragmatic. They include lexical re-signification (ironic uses of respectability labels), repositioning lived experience as evidence, small adjustments in relationship naming (“partner” rather than gendered labels), and bodily boundary-setting (hair, dress, sports participation). Small-business and craft narratives revalue domestic or informal work as economic agency. These moves open room for subjectivity yet remain entangled with traffic incentives and compliance rules, which constrain reach and durability. Visibility is also stratified: accounts serving professional, urban audiences achieve a wider radius for reinterpretation than those oriented to more tightly monitored or resource-constrained communities.

Taken together, the findings support three claims. First, mainstream discourses in the sample operate through a hybrid normalization: managerial reason and moral warmth, clinical neutrality, and individualized responsibility appear together to stabilize familiar roles. Second, agency is present but situated—most visible in small re-articulations that bend frames rather than overturn them, and most constrained where intersectional pressures (class precarity, non-heteronormative identities, migrant status) limit what can be said without penalty. Third, cross-platform references are best treated as contextual rather than civilizational contrasts. Differences in governance arrangements and market incentives across platform ecologies—within and beyond China—shape discursive possibilities in ways that resist simple binaries.

## Conclusions

This study used Feminist Critical Discourse Analysis (FCDA) to examine how gendered positions are articulated in a bounded corpus of WeChat Public Account texts. The contribution is analytic and context-specific: we identify a set of text-level mechanisms—lexical naturalization, obligation/permission marking, stance and voice arrangement, intertextual borrowing of authority, affective cueing, and strategic silence—and show how these mechanisms couple with account-level governance and distributional cues to make particular positions more sayable, visible, and legitimate. Rather than offering platform-wide generalizations, the analysis specifies how normalization and small re-articulations unfold in this environment, providing a transferable mechanism template that subsequent work can test across other modalities and settings.

Beyond these analytic contributions, the findings also offer implications for both digital feminist activity and policy communication. For digital feminist practices operating under platform constraints, the mechanisms identified in this study help explain why feminist expression on WeChat often takes adaptive, indirect, and strategically ambivalent forms. Recognizing how textual devices such as stance-taking, affective modulation, and strategic silence shape visibility may enable content creators, activists, and advocacy-oriented accounts to design communication strategies that remain legible within governance boundaries while still challenging naturalized gender hierarchies. For policymakers and institutional communicators, the analysis highlights how gender-related messages acquire legitimacy or produce unintended moralizing effects. Attending to the linguistic pathways through which gender norms are stabilized—including managerial vocabulary, moral warmth, scientized framing, and the individualization of structural burdens—can inform more inclusive and gender-responsive communication practices that foreground mutual respect, agency, and differentiated lived experiences.

Four limitations bound our claims. First, the sample is textual and platform-specific; images, video forms, and interactive affordances—common in short-video ecosystems—are outside our analytic window. Second, FCDA is optimized for production-side mechanisms and is less suited to estimating how audiences interpret or repurpose these texts. Third, our purposive design favors mechanism identification over prevalence, so we do not infer rates beyond the corpus. Finally, the findings are time-bound to a six-month window (June–December 2023); we therefore refrain from inferring seasonal or year-round trends.

Future work can extend both temporal scope and methodological breadth. Replication of the present protocol across adjacent semesters (e.g., January–June 2024) would help assess whether the discursive mechanisms identified here are stable over time or contingent on specific cycles of platform use and policy emphasis. Beyond timeframe replication, future research can expand both breadth and explanatory leverage. Cross-platform and multimodal analyses (e.g., short-video and livestream formats) can test whether the same textual cues align with visibility under different content pipes. Mixed-method designs—combining discourse analysis with audience reception studies and algorithm-adjacent evidence—would further illuminate how text-level mechanisms interact with distribution and uptake. Finally, intersectional sampling and pre-registered coding of mechanism families would facilitate comparative synthesis across studies and platforms and deepen understandings of gendered discourse in China’s evolving digital environment.

## Supporting information

S1 File English translations of the analyzed WeChat Public Account articles. This supporting file includes English translations of the 22 WeChat Public Account articles examined in this study. The file is provided for transparency and reference, enabling readers to better understand the textual data underlying the analysis.
(DOCX)
