# Supplementary material for: A feminist critical discourse analysis of gender norms on Chinese social media: Empirical insights from WeChat public accounts
Source: PLoS One. 2026 Jan 28;21(1):e0338967. doi: 10.1371/journal.pone.0338967 (PMC12851490; doi:10.1371/journal.pone.0338967)
Supplement: S1 File — This supporting file includes English translations of the 22 WeChat Public Account articles examined in this study. The file is provided for transparency and reference, enabling readers to better understand the textual data underlying the analysis. (DOCX) [file pone.0338967.s001.docx]

Article 1

Public Account Name:  [Gender studies horizon](javascript:void(0);)

Article Title: Gender mainstreaming: a strategic approach to safeguarding women's rights in the digital era

Date of Publication:  2023 June 21

Full Article Text:

In the digital age, protecting women’s rights faces new challenges. The dividends of digitalization do not automatically benefit everyone, as the gender digital divide and the negative impacts of digital technologies and tools constrain women’s development. To fully unlock the potential of frontier technologies for promoting gender equality and women’s empowerment, digital governance must integrate the strategies of human rights mainstreaming and gender mainstreaming, working to build a digital era that is inclusive, open, equitable, and shared.

The year 2023 marks the 30th anniversary of the *Vienna Declaration and Programme of Action*. Thirty years ago, this landmark document first recognized at the international level that “the human rights of women and girls are an inalienable, integral and indivisible part of universal human rights.” The assertion that women’s rights are human rights significantly shifted the marginalization of women’s rights at that time. Today, as we enter the digital era, safeguarding women’s rights faces new forms of challenge, requiring the continued implementation of gender mainstreaming strategies.

**01. Digital dividends do not automatically benefit all**

First, the gender digital divide hinders women’s equal participation. Existing inequalities combined with uneven digital development prevent women from equally benefiting from technological innovation. As UN Secretary-General António Guterres has noted, “3 billion people worldwide remain offline, most of them women and girls in developing countries; globally, women and girls make up only one-third of students in STEM; men outnumber women two to one in the tech industry, while women account for only about one-fifth of the workforce in AI; and only 3% of Nobel Prize laureates in the sciences are women.” In the poorest countries and regions, women are even more marginalized in digitalization; and within women as a group, rural women, older women, and women with disabilities face deeper exclusion.

The gender digital divide reflects entrenched stereotypes in the digital era. Many people firmly believe that men are inherently more technologically gifted, limiting women’s opportunities to access scientific education and participate in innovation. Some even argue that women’s engagement with technology and the internet is immoral, placing moral restrictions on their access. UN Women Executive Director Sima Sami Bahous has warned that the world faces “a form of digital poverty that is devastatingly excluding women and girls.” Without closing the gender digital divide, gender equality and sustainable economic recovery cannot be achieved.

Second, digital tools may entrench and exacerbate existing inequalities. Gender-blind digital governance models that ignore women’s specific needs fail to address structural bias and may even lead to indirect or systemic discrimination. As highlighted in the 2023 *Agreed Conclusions* of the UN Commission on the Status of Women (CSW67), digital technologies can indeed promote women’s rights, but they can also reinforce stereotypes and harmful norms, creating cycles where inequality is amplified and entrenched.

Big data, algorithms, and AI—often presented as neutral—are becoming conduits of gender discrimination. Male-dominated programming reinforces male perspectives while neglecting women’s needs. Datasets inevitably reflect existing social biases, underrepresenting women and marginalized groups. Algorithms designed or trained without gender awareness embed these biases into decision-making, further institutionalizing discrimination. Because algorithmic results are often perceived as objective and scientific, such biases become even harder to challenge, creating a gender divide more difficult to overcome than before.

Third, technology-enabled gender-based violence undermines women’s ability to benefit from digitalization. Online sexual crimes and trafficking, cyberstalking, harassment, bullying, remote sexual assault, hacking of women’s accounts and devices, theft and distribution of private information, non-consensual image manipulation, rumor-spreading, and hate speech are all proliferating. Young women—the most digitally connected generation—are the most vulnerable to discrimination and violence in online spaces. Tech-enabled gender violence is characterized by large-scale victimization, rapid spread, severe harm, greater concealment, and weaker accountability, posing new challenges for legal governance.

**02. Mainstreaming gender equality in digital decision-making**

A gender-blind approach to digital development runs counter to the goal of universal human rights. Gender mainstreaming has been recognized as a vital strategy for promoting equality and empowerment, and in the digital era, it remains central to national and international commitments.

**First**, gender perspectives must be integrated into digital decision-making. The *Vienna Declaration and Programme of Action* (1993) called for mainstreaming women’s rights across the UN system. In 1997, the UN Economic and Social Council defined mainstreaming as assessing the impacts of any policy, legislation, or program on women and men, making their experiences and concerns an essential dimension in all design, implementation, monitoring, and evaluation. The 2023 CSW67 *Agreed Conclusions* reaffirmed that the promotion and protection of women’s rights must be integrated into all policies and programs. Specifically, in the digital era, human rights must be safeguarded throughout the design, development, deployment, and regulation of technologies, while barriers to women’s equal access to science and innovation must be dismantled.

China has made progress in gender mainstreaming. The 2022 revision of the *Law on the Protection of Women’s Rights and Interests* introduced gender equality assessments of laws and regulations. In 2023, the Ministry of Science and Technology issued new rules encouraging awards for youth and women scientists. The draft *Regulations on Generative AI Services* (2023) explicitly prohibit discriminatory practices in algorithm design, training, and outputs based on gender, ethnicity, or nationality. These measures reflect efforts to embed gender perspectives in digital governance.

**Second**, the digital era has stimulated women’s agency. In 2015, the UN General Assembly established the International Day of Women and Girls in Science (February 11) to promote their participation. The 2023 CSW67 called on states to ensure women’s full and equal participation in technology and innovation leadership and employment. China has sought to unleash women’s potential in STEM. According to WIPO, Chinese women account for 24% of international patent applications, higher than the global average of 17%. Between 1982 and 2017, the number of female technical professionals in China rose from 10.1 million to 15.3 million, with women’s share increasing from 38.3% to 47.8%. Today, women account for 55% of internet entrepreneurs in China, reshaping digital gender norms and encouraging collaboration with men.

**Third**, gender equality and digital education empower women and girls. The 2019 “Beijing Consensus on AI and Education” called for gender-sensitive AI applications in education. In 2021, UNESCO released *AI and Education: Guidance for Policymakers* and later adopted the *Recommendation on the Ethics of AI*, naming gender as a priority policy area. The 2023 CSW67 further urged countries to advance gender equality in digital and STEM education. In China, gender gaps in compulsory education have been nearly eliminated, and girls have comprised over 50% of general high school students for seven consecutive years. However, gender preferences in higher education remain marked, particularly in STEM. To eliminate women’s disadvantages, efforts must begin early, nurturing girls’ scientific awareness and innovation skills.

In the digital era, the notion of technological neutrality can no longer be taken for granted. To fully unleash the transformative potential of frontier technologies for gender equality and women’s empowerment, digital governance must adhere to the strategies of human rights mainstreaming and gender mainstreaming, working to build a digital environment that is inclusive, open, equitable, and shared.

Article URL (optional): <https://mp.weixin.qq.com/s/iROCrGA5Nnp3Qn8kuWYMbg>

---

Article 2

Public Account Name:  A green apricot

Article Title: Not wanting a daughter—is that misogyny?

Date of Publication:  2023 June 26

Full Article Text:

Some time ago, during a gathering with friends, my close friend P announced that she was pregnant.

We all knew she and her husband had been trying for a long time, so we congratulated her and wished her dream come true. She had often said before that she hoped for a daughter.

To our surprise, this time P shook her head and said she had changed her mind: *“I always wanted a daughter, but once I actually became pregnant, I realized I’d rather have a son.”*

I was startled—P had never shown any preference for sons over daughters—so I asked her why she had changed her view.

She counted on her fingers: *“Think about it—boys don’t have to endure monthly periods or menstrual pain; they won’t suffer morning sickness or the agony of childbirth; they won’t have dietary restrictions during pregnancy or breastfeeding; they won’t face added workplace pressure due to fertility; and they won’t lose as much personal freedom after becoming parents. It’s not that I value sons over daughters. I genuinely love girls, but I don’t want my daughter to go through all that pain. That’s why I hope for a boy.”*

Of course, wanting a child of a particular gender is a matter of personal choice, and the baby’s sex is only revealed at birth. Regarding P’s anxiety, as friends we could only comfort her and accompany her more to ease her stress.

Yet after returning home, I kept thinking about what P said. On the surface, her reasoning came from love for a daughter, but something felt wrong. I searched online and found that many women share the same mindset.

One short video titled *“I really don’t dare to have a daughter”* had 59,000 likes. Comments read: *“I don’t want to worry about her marrying far away and being mistreated,”* *“Women are a disadvantaged group—since they must suffer childbirth, better to have a boy. Men just need to earn money.”*

At that moment, I suddenly realized: women who *“don’t want daughters because they love daughters”* are, in fact, rejecting their own fate as women. This is another form of misogyny.

As scholar Chizuko Ueno explained in *Misogyny*, the phenomenon manifests differently in men and women. For men, it appears as contempt for women; for women, it shows as self-hatred—resentment of their bodies, menstruation, or the painful process of childbirth. Men say, *“Luckily I’m not a woman,”* while women lament, *“If only I weren’t female.”*

Honestly, I too have had such moments growing up. At school, whenever menstrual pain disrupted my classes. At university, when I wanted to major in forensic science, but my father stopped me: *“If you, a girl, choose this field, who would marry you? If you insist, don’t come back—I’ll consider you no daughter of mine.”* Two years after graduation, I wanted to use my savings and borrow 200,000 yuan from my father to make a down payment on an apartment. He refused, saying: *“Why does a girl need property? Keep your savings for us until you marry, otherwise it will become marital property.”* He later lent the money to a male relative for his business, explaining: *“A man needs a career, or he can’t marry.”*

Countless times, I thought bitterly, *“If only I weren’t a girl.”* I blamed my gender as the source of injustice.

But now I see—I had fallen into the same trap as those women who prefer sons. Women are quick to perceive the unequal burdens assigned to men and women in a patriarchal society and the injustices rooted in gender difference. Yet, constrained by social norms and self-limitations, many unconsciously accept patriarchal logic, internalize misogyny, and resent their own sex—believing discrimination against women is inevitable.

Take a simple example: many such women worry that daughters must endure pregnancy, breastfeeding, and childcare. But why assume a daughter *must* give birth? Why presume breastfeeding is obligatory? Why can’t motherhood be a voluntary act of love and commitment, instead of a compulsory burden imposed by patriarchal norms?

The liberation of mothers and children is closely tied. A mother who accepts patriarchal discipline passes it on to her children: sons may grow into misogynistic men, while daughters internalize their parents’ misogyny and live in self-hatred.

Feminists are those who recognize their own internalized misogyny and consciously fight against it. If even women despise their own sex, how can they love themselves? And without self-love, how can women live joyfully and fully?

The injustices and burdens women face are not the fault of their sex, but of a discriminatory system and those who uphold it.

As women, what we can do is refuse to internalize the ideology of female inferiority, learn to accept and love our gender, fight for every instance of equality, and ultimately break the vicious cycle of misogyny—so that a truly equal world may emerge.

Article URL (optional): <https://mp.weixin.qq.com/s/LtVj1-PiQif8ZZZCyYp2_Q>

---

Article 3

Public Account Name: A green apricot

Article Title: After three months of struggle, I finally cut my hair Short

Date of Publication: 2023 July 03

Full Article Text:

“Hello, I’d like to cut my hair this short,” I said to the stylist Tony, pointing to a photo of the female lead in *Wolf Warrior 2* on my phone.
“Are you sure?” he asked, glancing at me and then at the photo. I nodded firmly. He confirmed several more times before finally making the first cut.

When did I first want to try a buzz cut? Probably back in high school. At the time, academics were demanding, and we had only half a day of rest each week. Since the school didn’t provide hot showers, we had to carry kettles to the canteen for hot water. My thick, frizzy hair was so difficult to manage that the only way to control it was to keep it long and tied up. Yet that also meant extra hassle: more hot water, more time, and unbearable summer heat when my hair took forever to air-dry—since blow dryers weren’t allowed.

Gradually, I came to see my hair as a burden. Boys with short hair could rinse off quickly after basketball, while every stylist who cut my hair marveled, *“You have at least twice, no, three times the average hair volume!”* Family members also often complained about hair everywhere in the house. Even if unintentional, these remarks left a deep scar on my young mind. At that age, I lacked the courage to defy others’ opinions by cutting my hair short, let alone shaving it close.

After starting work and cleaning my own apartment, I found hair everywhere—on the pillow, on the floor. Influenced by my mother, I developed a mild obsession with cleanliness, and stray hairs bothered me endlessly. I tried short haircuts, but my frizz meant I needed hair-straightening every two months; otherwise, I looked like a lion. After going through this cycle a few times, my desire for a buzz cut grew stronger. Every morning as I struggled with my hair, the thought returned. Eventually, when it was time to straighten my hair again, I made the decision to cut it all off.

And so, amid the buzzing of the clippers, a new look was born (not bald, but about 15mm of hair left). Looking in the mirror, I saw a self both familiar and new. Shaking my head, I felt lighter, freer.

At home, I examined my new head closely. My skull wasn’t perfectly round; the crown was pointed, the back somewhat flat. Yet I loved it, and couldn’t stop touching my hair. At last, nothing blocked the “cooling system” of my fast-running brain—I joked that its performance would only improve from here.

Of course, there were social impacts. The first day I went to work with my new haircut, I wore a hat, nervous about colleagues’ reactions. Over lunch, I told them with a smile, “I cut a buzz cut—want to see?” They were visibly surprised, some couldn’t help glancing at my head during conversation, and close friends asked to touch it. For a while, I debated whether to keep wearing hats. Luckily, within a couple of weeks, the curiosity faded.

When I visited my parents, I also wore a hat, hesitant to shock them. I told my mother in advance: *“I cut my hair short—it’s not because of heartbreak or illness. It’s just too much trouble, so I wanted to try something new.”* When they saw it, their eyes betrayed disapproval: *“Why so short? Like a boy’s haircut. Grow it back.”* At first, they often brought it up, but gradually less so. One day, to my surprise, my mother asked, *“Do you think I’d look good with short hair?”* I suddenly realized—my thick hair had come from her. Though thinner with age, it was still far above average. Guess what? She eventually tried a short cut herself.

Overall, the benefits outweighed the discomfort of others’ reactions. Washing my hair became quick and easy, with no hair all over the house. Mornings were simpler—I could be out the door in ten minutes. I also spent less energy on makeup and dressing up, not because the haircut changed me, but because I finally felt free to let go of expectations shaped by culture. I now choose clothes for comfort and invest more time in things I enjoy.

Of course, I’m not suggesting every woman should get a buzz cut. What I hope for is more tolerance in society: boys can like skincare and long hair; girls don’t have to fit into the mold of “fair, slim, and delicate” beauty. They can wear short hair—or even a buzz cut—if they wish. Everyone should have the freedom to choose how they present themselves.

Article URL (optional): <https://mp.weixin.qq.com/s/Fnr0ECB211wOfRr4v4bO7Q>

---

Article 4

Public Account Name: Gender studies horizon

Article Title: What factors should be considered when returning the bride price?

Date of Publication: 2023 December 12

Full Article Text:

Issued on December 11 by the Supreme People’s Court (SPC), the Ministry of Civil Affairs, and the All-China Women’s Federation

Case 1

Where the parties have registered their marriage but cohabited for a short period, an excessively high bride price should be partially returned at divorce, taking into account cohabitation length, pregnancy/childbearing, and other facts.

I. Basic Facts

In September 2020, Wang married Li (female) by registering their marriage. Wang’s family was low-income locally. To facilitate the marriage, Wang paid Li a bride price of 188,000 yuan. In April 2021, Li terminated a pregnancy. As conflicts between the families intensified, Wang filed for divorce in February 2022, also seeking return of the full 188,000 yuan bride price.

II. Judgment

The court found that the parties lacked sufficient acquaintance before marriage and failed to build a solid relationship afterward, leaving no basis to maintain the marriage; divorce was granted. Given local living standards and Wang’s financial situation, the 188,000 yuan constituted an excessively high bride price that imposed a substantial burden. Considering the short cohabitation period and the fact that the woman had terminated a pregnancy, the court, to balance interests and resolve the dispute, ordered Li to return 56,400 yuan.

III. Significance

A bride price is property given pursuant to custom for the purpose of concluding a marriage. While it conveys affection and expectations for the union, excessive payments beyond one’s means distort the essence of marriage into material exchange, impose economic pressure on the payer, and undermine family harmony and social civility. Since 2021, the “No. 1 Central Document” has, for three consecutive years, called for addressing exorbitant bride prices, reflecting broad social consensus.
Generally, where the parties have registered their marriage and cohabited, courts will not support a claim to return the bride price upon divorce. However, because the purpose of paying a bride price includes not only completing the legal formality of registration but, more importantly, establishing long-term cohabitation, the duration of cohabitation should be a key factor in deciding whether and to what extent a return is warranted.
Here, the parties lived together for only a little over one year; the purpose of the bride price was not fully realized; the payer bore no obvious fault; the amount was high relative to the family’s income and caused a heavy burden. Considering also the physical impact of pregnancy termination on the woman, ordering a partial return appropriately balanced the parties’ interests, guided sound views on marriage, and promoted frugal, civil wedding customs—so that marriage begins with love and the *caili* returns to the realm of “li” (proper rite).

Case 2

Where the parties held a wedding ceremony, cohabited for a long time, and have children, courts generally will not support returning the bride price.

I. Basic Facts

Zhang and Zhao (female) met through introduction in November 2018, began cohabiting in February 2019, and had a son in June 2020. In January 2021, they held a wedding ceremony but never registered their marriage. Zhao received 160,000 yuan as bride price from Zhang. After the relationship broke down, they ended cohabitation in August 2022. Zhang sued, seeking return of 80% of the bride price (128,000 yuan).

II. Judgment

The court found the parties had cohabited since February 2019 and held a customary wedding; they had a son who had already turned two. Cohabitation entails daily expenses and costs related to childbirth and childrearing. Demanding return of the bride price after years of living as husband and wife in fact, and having jointly raised a child for two years, would be manifestly unfair to Zhao. The claim was dismissed.

III. Significance

General Secretary Xi Jinping has emphasized the foundational role of the family and the importance of family education and virtues. The Civil Code calls for cultivating good family traditions, promoting family virtues, building family civility, and protecting the lawful rights and interests of women, minors, the elderly, and persons with disabilities.
In hearing bride price disputes, courts must implement these principles and relevant provisions of the Civil Code. Article 5 of the SPC’s *Interpretation (I) on the Marriage and Family Book of the Civil Code* provides for return of a bride price where no marriage registration was completed, but this should be limited to situations where the parties did not cohabit. Even without legal spousal status due to lack of registration, the “substance of a marital relationship” created by cohabitation should not be overlooked. Such cohabitation advances the payer’s central purpose and may affect the woman’s physical and mental health, especially when children are involved. Ordering full return solely because there was no registration would contravene fairness and be detrimental to protecting women’s rights.
Here, though unregistered, the parties held a customary wedding, cohabited as a couple for over three years, and had a child. The judgment accords with local customs, balances interests, and particularly safeguards women’s lawful rights.

Case 3

Where marriage registration occurred but only brief cohabitation followed without forming a stable common life, a partial return of the bride price is appropriate after deducting joint expenditures.

I. Basic Facts

Liu and Zhu (female) began dating in July 2020 and registered their marriage in September 2020. That month, Liu transferred 800,000 yuan to Zhu’s bank account with the note “bride price,” and another 260,000 yuan marked “five golds” (traditional jewelry). They lived and worked in different provinces. Disputes arose over wedding preparations, and they divorced by agreement in November 2020—less than three months after registering. They had no children, no joint property, and no joint debts. They briefly cohabited and incurred costs for wedding preparation, wedding photos, travel, and family interactions. After divorce, Liu sued to have Zhu return 1.06 million yuan.

II. Judgment

The court held that the amounts marked “bride price” (800,000 yuan) and “five golds” (260,000 yuan) both fit customary understanding of bride price and should be treated as such. Although the parties had registered their marriage, they were still in the process of holding a customary ceremony. The marriage lasted less than three months; they lived in different cities and had no shared plan for future work, residence, or life. Their brief cohabitation did not form a complete family unit or stable common life.
Given the registration, and that after payment of the bride price the parties jointly prepared the wedding, traveled, and had reciprocal family visits incurring shared expenses, those expenses should be deducted. The court therefore ordered a return of 800,000 yuan.

III. Significance

In disputes over returning bride price—regardless of whether the parties registered their marriage—the length of cohabitation is a key factor in deciding whether and how much to return. Because circumstances vary, there is no rigid standard for “cohabitation”; it must be assessed case by case.
Here, the marriage lasted a short time; after registration, the parties were still preparing the ceremony; they lacked a unified plan for future life and did not form a stable common life. Thus it was inappropriate to deem them as having cohabited in a stable sense. Considering the impact on the woman of registration and brief cohabitation, the existence of shared expenditures, and the excessive amount, ordering a substantial partial return appropriately balanced interests.

Case 4

In disputes over engagement property, the parents of the recipient of the bride price may be listed as co-defendants.

I. Basic Facts

Zhang (male) and Zhao (female) met through introduction and became engaged in April 2022. Zhang paid 36,600 yuan in betrothal gifts to Zhao’s parents, Zhao Sr. and Wang; in September 2022, he transferred 136,600 yuan in bride price to Zhao’s bank account. Zhao purchased dowry items worth 1,120 yuan and placed them at Zhang’s residence. The parties neither registered their marriage nor held a wedding ceremony. After the engagement was dissolved in September 2022, Zhang sued, seeking joint return of 173,200 yuan from Zhao and her parents.

II. Judgment

The court found there was no marriage registration and insufficient evidence of continuous, stable cohabitation. Zhang bore no obvious fault, but the evidence showed Zhao also made efforts toward marriage. Therefore, after deducting the dowry value, an appropriate return was warranted.
As to proper defendants: the 136,600 yuan bride price was directly transferred to Zhao and should be returned by Zhao; after deducting the dowry, 121,820 yuan was ordered returned. The 36,600 yuan in betrothal gifts was jointly received by Zhao and her parents; accordingly, Zhao and her parents were jointly ordered to return 32,940 yuan.

III. Significance

Article 10 of the Civil Code provides that civil disputes are to be handled according to law; where the law has no provision, custom may be applied provided public order and good morals are not violated. As there is no statutory rule specifically governing bride price, courts should handle such disputes in accordance with custom without breaching public policy.
Under traditional custom, the giving of a bride price in the engagement process is negotiated and participated in by both sets of parents, often with relatives and matchmakers as witnesses. This should be considered when determining the proper parties to a lawsuit. Practices vary widely across regions and families: the recipient may be the engaged party, the parents, or both; the funds may become part of the dowry, be returned to the couple as start-up funds, or be otherwise used by the recipient’s family. Where a party’s parents received the bride price, their receipt may be deemed a joint act with their child; listing both the engaged party and the parents as co-defendants accords with custom and facilitates fact-finding on the amount and use of the bride price, enabling lawful adjudication.

Article URL (optional): <https://mp.weixin.qq.com/s/gOkwViCoYxQlLevF6whwUA>

Article 5

Public Account Name: Gender studies horizon

Article Title: Supreme court and all-China women's federation release model cases of domestic violence crimes

Date of Publication: 2023 November 25

Full Article Text:

Since the 18th National Congress of the Communist Party of China, the CPC Central Committee has attached great importance to the construction of family civility. General Secretary Xi Jinping has stressed: *“Only when every family is well, can the country be well; only when every family is well, can the nation be well.”*

The family is the basic unit of society. Harmonious and stable family relationships not only contribute to individual happiness and the healthy growth of minors, but also form a solid foundation for national development, social progress, and social harmony. Domestic violence is a malignant tumor in society: it causes grave physical and psychological harm to victims, negatively affects children’s development, may escalate into criminal cases, and seriously undermines social stability and the people’s sense of security, happiness, and fulfillment.

People’s courts firmly oppose domestic violence, giving full play to their adjudicative functions. They punish domestic violence crimes in accordance with the law; for crimes such as intentional homicide and intentional injury that gravely violate personal rights, heavy sentences—including the death penalty where appropriate—are imposed to safeguard victims’ lawful rights and interests.

Women’s federations, as the “bridge” between the Party/government and women, and as the “maternal family” of women, attach great importance to and actively engage in anti-domestic violence work. They provide services such as psychological counseling, legal consultation, assistance with police reports, proxy applications for personal safety protection orders, and care for victims in hardship. Courts and women’s federations complement each other’s strengths, cooperating to: identify domestic violence cases early, provide timely intervention and diverse forms of assistance for victims and minors, carry out legal education, and promote family civility. These efforts have achieved remarkable results.

November 25 is the International Day for the Elimination of Violence Against Women, also known as the International Day Against Domestic Violence. To raise public awareness, safeguard the rights of victims—especially women and children—and deter perpetrators, the Supreme People’s Court and the All-China Women’s Federation jointly released five typical criminal cases involving domestic violence.

These cases cover crimes such as intentional homicide, intentional injury, and abuse. The victims include both family members and cohabitants outside the legal family. The cases span the most common forms of domestic violence (“beating wives,” “beating children”), as well as homicides triggered by domestic violence and violations of personal safety protection orders.

The cases reflect four guiding principles:

1. Fully Implement the Policy of Combining Leniency with Severity

Punishing domestic violence crimes must follow the principle of tempering justice with mercy. Such cases should not be treated more leniently merely because they occur within families, nor should they all be treated with blanket severity. The punishment must fit the crime. For perpetrators who use cruel means, cause severe consequences, or commit violence due to vices such as alcoholism, drug abuse, or gambling, the law requires strict punishment. For example, in the released case of defendant Xie, who committed intentional homicide, the crime was extremely serious and the death penalty was imposed and approved. For victims who, after suffering long-term domestic violence and severe physical and psychological harm, retaliate by injuring or killing the abuser, courts should consider the cause, nature of the act, and the abuser’s fault, and handle such cases with leniency. For instance, in the case of defendant Zhao, the court recognized her crime as “relatively minor” and imposed a lighter sentence.

2. Encourage Victims to Seek Legal Protection

Thanks to the Anti-Domestic Violence Law and broader legal education, the view that domestic violence is not merely a “private family matter” has gained wider acceptance. Nevertheless, misconceptions remain, and some victims, influenced by ideas such as “family shame should not be made public,” hesitate to seek help, preventing timely intervention. In the case of defendant Liang, the victim took the initiative to call the police and sought help from the local women’s federation, using legal and social resources to free herself from abuse—setting a positive example for other victims to bravely say “no” to violence. Conversely, the case of defendant Zhao, who killed her abusive partner, serves as a reminder that victims must protect their rights through legal channels. Resorting to crime only leads to imprisonment and hardship for themselves and their families.

3. Defend the Judicial Authority of Personal Safety Protection Orders

Since the Anti-Domestic Violence Law came into effect on March 1, 2016, the system of personal safety protection orders has been established. The SPC has issued judicial interpretations clarifying evidentiary standards and adjudication rules, and, together with the ACWF, released guidelines for strengthening implementation. The issuance rate of protection orders has steadily increased, providing a strong safeguard against violence.
However, some perpetrators still fail to respect or blatantly violate protection orders, undermining their effectiveness and judicial authority. For example, in the case of defendant Wang, who was prosecuted for refusing to comply with a ruling, the violation of a protection order led to criminal liability—sending a strong signal that such orders are not “mere pieces of paper” and violations will be punished.

4. Emphasize Prevention and Assistance

Anti-domestic violence efforts must prioritize prevention, timely intervention, and effective protection, requiring active participation and coordination across all sectors. Families should foster mutual respect, rationally resolve disputes, and reduce violence at its source. Community-level organizations (residents’ committees, village committees), public security organs, and mediation committees should strengthen early detection of risks and provide timely intervention. Relevant departments should further improve shelter, medical, aid, and guardianship measures to help victims and families escape the shadow of violence as soon as possible.

“The foundation of the state lies in the family.” Family harmony is the cornerstone of social civility. Combating domestic violence is the shared responsibility of the state, society, and every household. The call is for all sectors and citizens to raise awareness, take active measures, and jointly foster a new ethos of socialist family civility characterized by mutual love and respect.

Article URL (optional): <https://mp.weixin.qq.com/s/bsXh7k5QZfmTxd9C9Gs3og>

---

Article 6

Public Account Name: Gender studies horizon

Article Title: New Occupations and Business Models Drive New Developments in Women's Employment

Date of Publication: 2023 July 21

Full Article Text:

China’s digital economy is booming. In areas such as artificial intelligence and autonomous driving, new technologies are at the forefront globally. Emerging sectors and models such as express delivery, food delivery, and internet-based healthcare have created hundreds of millions of flexible jobs. The *BRICS Women’s Development Report (2023)* shows that China’s digital economy accounts for nearly 40% of GDP and has created 57 million jobs for women.

How should we understand women’s quality employment in these new sectors, the enhancement of women’s skills, and how to avoid “race-to-the-bottom” competition? A journalist from *China Women’s News* conducted an in-depth interview with Zhang Bingzi, Director of the Population and Employment Research Office, Department of Social and Cultural Development, Development Research Center of the State Council.

Q: At present, new forms of employment have broken through the rigidity of traditional employment, showing greater flexibility, mobility, variability, and inclusiveness. From the supply side, where do you see the “new” aspects?

Zhang Bingzi: New sectors and the flexibilization of the labor market have provided workers with new employment choices, which can be summarized in two main ways: More flexible forms of employment. In terms of work location, people can now work remotely and online. In terms of work time, they can choose flexible schedules, short-term contracts, or gig work, gaining much greater freedom in time and place. New work fields. With breakthroughs in next-generation ICT, AI, biotechnology, new materials, and new energy—combined with traditional industries—more job opportunities and fields have emerged, transforming traditional work content.

Q: The contribution of new forms of employment to women is not only in scale, but also in quality and effectiveness. How do you understand the view that “new sectors promote high-quality employment for women”?

Zhang Bingzi: First, women now have more choices in employment. Second, flexibility in time and place offers opportunities to many women who cannot work full-time. Third, the rise of internet and online platforms provides relatively fair and open channels, lowering barriers to entrepreneurship and freelancing, and thus expanding opportunities.

Especially with the growth of the consumer internet, daily consumer goods have proliferated, and consumer experience has become central to competitiveness. Since women are the primary decision-makers for personal and household consumption, women workers are uniquely positioned to understand and meet female consumers’ needs—offering a clear advantage in market competition.

Q: At the same time, women face growing employment challenges in these new sectors. What gender-related difficulties are most evident?

Zhang Bingzi: Despite changes in some traditional employment features, the disadvantages women face in the labor market remain. Unpaid care work persists. Women still bear the main burden of household chores, childcare, and eldercare. Flexible schedules do not remove the “double burden,” but may shift the expectation of balancing work and family entirely onto individuals, obscuring systemic gender inequalities. Gender stereotypes remain. These limit women’s development in emerging industries and can even be embedded into algorithms. Since these industries are science- and technology-intensive, stereotypes that women lack ability or interest in STEM hinder their education, employment, and promotion opportunities. Algorithms trained on biased historical data may reproduce such inequalities in hiring, promotion, and pay. Platforms may reinforce stereotypes. Personalized recommendations and advertising often target users by gender, interests, or habits, further entrenching gender stereotypes and even exacerbating gender tensions.

Q: With rapid growth, problems such as unclear labor relations and weak rights protections have become prominent. What specific risks do women workers face in these uncertain employment contexts?

Zhang Bingzi: Flexible employment has exposed inadequacies in labor rights protections. For women, risks may be magnified.

In traditional employment, rights such as occupational safety, income security, working hours, social insurance, and benefits are institutionally safeguarded. Under flexible arrangements, many work as freelancers, gig workers, or contractors, outside the protections of traditional labor relations. As women’s participation in flexible work rises, so too do risks in economic security and workplace safety.

Q: Many new sectors form labor boundaries through technology. How can women enhance competitiveness, avoid “race-to-the-bottom” dynamics, and achieve fuller, higher-quality employment?

Zhang Bingzi: To enhance women’s competitiveness, both skill improvement and supportive policies are essential. Key areas include: Education and training. Promote digital skills and lifelong learning. Encourage girls to enter STEM fields, with teachers trained in gender equality awareness. Strengthen career guidance, providing women with digital career information and pathways. Human resource service companies should align training with market demand; platform enterprises can offer online learning platforms; professional institutions should provide practice-oriented courses to improve applied skills and problem-solving. Maternity insurance. Reduce burdens for both women and their employers. Maternity insurance provides medical services and income replacement during leave, ensuring women’s income without imposing higher costs on employers. This reduces discrimination against firms employing women and promotes equality in family and labor roles. Measures include paying maternity benefits from insurance funds (not employers), and introducing paternity leave to encourage men’s childcare participation. Childcare services. Expand public childcare systems, improve standards and convenience, and raise utilization. Services should be affordable, reliable, and accessible—including full- or half-day care for infants, temporary childcare, and after-school programs for school-age children. Labor rights protection. Address excessive working hours and reduce the costs of defending against discrimination. Strengthen legislation to penalize overwork, enforce regulation, and improve anti-discrimination laws. Establish dedicated institutions to lower the cost of rights protection for vulnerable workers, particularly women with heavy caregiving responsibilities.

Article URL (optional): <https://mp.weixin.qq.com/s/A63ILo5LGm86c0LlX7lKsQ>

---

Article 7

Public Account Name: Gender studies horizon

Article Title: Unlocking the power of gender equality to create infinite possibilities

Date of Publication:  2023 September 18

Full Article Text:

Gender equality is a fundamental marker of social progress and an essential dimension of high-quality population development. Sustained advancement in gender equality contributes to the recovery and improvement of fertility levels.

Population is a foundational and strategic variable in economic and social development. At present, the world is undergoing profound changes unseen in a century, as well as a demographic transformation of equal magnitude. In 2022, the global population reached 8 billion, accompanied by major shifts in population patterns—such as slower growth rates and shifts in the geographic distribution of population.

China’s demographic development has also entered a “new normal,” marked by population decline, aging, low fertility, and regional disparities in population change. At the same time, China’s population will remain above one billion for the long term. The Report to the 20th National Congress of the Communist Party of China identified “a huge population size” as the first defining feature of Chinese modernization. While such scale brings pressure in terms of services and support, it also provides strong momentum for development.

The Party leadership has emphasized that advancing high-quality population development is essential for supporting Chinese modernization. This means focusing not only on population quantity, but also on improving education, health, employment, and income—closely linking high-quality population development with a high-quality life for the people, promoting holistic human development and common prosperity.

Among demographic challenges, responding to low fertility rates is the core and most difficult issue. Women are central to fertility. International experience shows that sustained progress in gender equality helps restore and raise fertility levels. From the history of women’s development in China, it is clear that the empowerment and advancement of women profoundly reshape economic and social progress as well as family life patterns.

Gender equality is both a basic measure of social progress and one of the United Nations’ Sustainable Development Goals (SDGs) for 2030. It is also a vital dimension of high-quality population development. However, according to the *Global Gender Gap Report 2023* released by the World Economic Forum, global progress toward gender equality is slowing. While gender gaps in education are narrowing, inequalities in labor market participation and other areas remain worrisome.

China has long committed to promoting gender equality, enshrining it as a basic national policy, and has made tremendous achievements in women’s development. Yet, as a large developing country emerging from a semi-colonial, semi-feudal past, China still has much room for improvement in gender equality in employment, the economy, and political participation. After the adjustment of fertility policies, parents now face the responsibility of raising multiple children, and women in particular confront new challenges and pressures in balancing childbearing, career development, and family care.

Only by addressing women’s difficulties in reproduction, career progression, and family caregiving can families be better supported in realizing their fertility intentions—thereby promoting family harmony and happiness, maintaining a moderate fertility level, and advancing human freedom and comprehensive development.

The theme of this year’s World Population Day was: “Unleashing the Power of Gender Equality: Uplifting the Voices of Women and Girls to Unlock Our World’s Infinite Possibilities.” Indeed, only when women—who make up half the world’s population—are fully integrated into development can we achieve a fairer, more inclusive, and sustainable future.

Against the backdrop of China’s new demographic normal, it is imperative to further empower women, fully tap the gender dividend, and advance both population and economic development in high-quality ways. When women face no discrimination in employment, education, and income, and when they possess decision-making power and autonomy, only then can they realize free and comprehensive development.

Article URL (optional): <https://mp.weixin.qq.com/s/6hCqt-yvsAzoiQjnWlq2iQ>

---

Article 8

Public Account Name: A green apricot

Article Title: Can we move beyond the supremacy of sexual attraction?？

Date of Publication:  2023 November 08

Full Article Text:

The term *“xingyuan relationship”* essentially refers to intimate relationships such as romantic love; while *“xingyuan brain”* describes a mindset in which one views such relationships as the most important factor in human interaction. For instance, upon meeting someone of the opposite sex, one immediately evaluates whether they could become a future partner; or when attracted to someone, one devotes all attention to that person while neglecting others and other aspects of life. This narrows perspective, eclipses other possibilities, and distorts normal interactions.

To put it simply, *“xingyuan brain”* means *placing romance above all else*—upholding the supremacy of love, as if other kinds of relationships should be sidelined.

I can list many of my own “glorious deeds” under the spell of this mindset. For example, when I was in my first year of high school, during a psychology class the teacher asked: *“What do you think is the most important thing in life?”*

When it was my turn, I stood tall and loudly declared before the whole class: *“Teacher, I believe the most important thing in life is love.”*

The teacher pressed: *“Would you be willing to sacrifice everything for love?”*
I answered: *“Yes, I would sacrifice everything.”*

She asked again: *“Does ‘everything’ include your very life?”*
I again said: *“Yes.”*

At that, my classmates gasped in unison. I even heard them whisper: *“Wow, whoever marries her will be so lucky!”*

The teacher said no more, but let me sit down. Outwardly I was calm, but inside I was thrilled. I thought: *“I am willing to sacrifice everything for love. Even my classmates admire my courage.”*

What I did not realize was that I had already placed romantic love on a pedestal and chained myself in the shackles of *love supremacy*—becoming, in fact, a supporter of romantic absolutism.

Time passed. Though I had not yet encountered the actual term *“xingyuan brain”*, I began to sense what it meant.

Once, when I was working on a project with a male classmate and found the collaboration pleasant, I casually praised him. Somehow word spread, and soon I was teased constantly: *“You must like him—why else would you compliment him?”*

Whenever he appeared alone, or we were both present, classmates would make suggestive remarks. No matter how I explained, they either ignored me or smiled knowingly: *“Explanations are just cover-ups—we all know you like him.”*

This left me embarrassed and frustrated. I thought: *“Why must they interpret everything as love? Complimenting someone doesn’t mean I like him romantically!”*

Reflecting further, I realized my discomfort came not only from the violation of my personal boundaries, but also from a change in my own perspective. I began to see that relationships between women and men need not be confined to romance. Nor is love the entirety of life. So why must my classmates erase other possibilities and force everything into the frame of love?

Romantic relationships have their significance, of course, and I do not deny love’s value. But elevating it above all else—turning it into a form of hegemony—is unreasonable. Alongside love, we also have family ties, friendship, teacher–student bonds, and other diverse emotional experiences that are just as precious.

So why do we ignore these rich forms of connection and single out only love, lifting it to a divine status? Such a life is not only monotonous, but absurd.

In the end, my wish is this: that we become aware of this tendency and learn not to live so utterly bound by *love supremacy*. May we open our eyes to other feelings and other possibilities, enriching our lives beyond the narrow confines of romance.

Article URL (optional): <https://mp.weixin.qq.com/s/OFSXkoPnFy1jorIvMq2FEw>

---

Article 9

Public Account Name: A green apricot

Article Title: After a year of gender-free dating, I turned my boyfriend into a “Girlfriend”

Date of Publication: 2023 June 14

Full Article Text:

First, let me clarify: there is no academic definition of *“genderless love.”* This term was something my boyfriend and I coined on a whim.

It started one day when I noticed that some of his thoughts struck me as rather “feminine.” Looking at him more closely, with his slim, delicate frame, I couldn’t help blurting out: *“If only you were my girlfriend.”*

Startled, he asked: *“Do you mean you’re actually homosexual?”*
“No, no, no,” I hurried to explain. “I can’t quite describe it—I just suddenly felt that you seemed like a cute girl, and I wanted to treat you the way I’d treat a girlfriend.”

At the time, I couldn’t fully parse the fleeting thought, but I sensed it was connected to wanting to break through gender stereotypes in an intimate relationship. So, half-jokingly, I coined the phrase *“genderless love.”*

Here I must praise my dear boyfriend. Unlike some men who carry obvious misogyny, he took being called “like a girl” as a genuine compliment, even feeling happy about it. More than that, he was willing to help me explore my fuzzy ideas.

Yet, though my original intention was to challenge stereotypes, when I first began to “practice” this idea, I ironically reinforced them. I wrongly interpreted “treat him as if he were a girl” as meaning “act like a stereotypical boyfriend toward him.” So I exaggerated the “masculine” side of myself, even searching on lifestyle apps for “model boyfriend behaviors” and following them one by one.

On rainy days, I messaged him asking if he had brought an umbrella, offering to pick him up—even though I knew he always carried one, and realistically, as a graduate student while I was already working, I couldn’t commute to his campus just to escort him. On weekends, when he visited my home—a ten-minute walk from the subway—I insisted on meeting him at the station, as if those ten minutes would be unbearable for him. At home, I barely let him move from the couch, handing him food and drinks, nearly doing everything for him—short of following him into the bathroom.

Naturally, I earned his exasperated response: *“Sis, are you treating me like a girl, or like a disabled patient?”* Then came the sobering question: *“Would you want me to treat you this way?”*

Of course not. I want to be treated as an equal, with respect and understanding, and to be recognized and celebrated when I achieve something through my own effort—not to be patronized like a child. How could I have confused such dependency with “treating a girl well”?

What I had really meant, at the beginning, was: *“I accept that you are a man who also embodies traits society labels as feminine. I don’t want to correct or restrain you with gender norms.”* But stereotypes are pervasive and insidious. In trying to escape the trap of *“men must be like this,”* I fell into the trap of *“women must be like that.”*

After serious conversations, my boyfriend and I decided to keep the lofty name *“genderless love”* and continue exploring what it means in practice. After all, the purpose of entering an intimate relationship is to create a shared space where we can temporarily shed society’s constraints and safely be the selves that society does not always accept.

We even adjusted our language. For example, when my boyfriend once wore a skirt to a comic convention, he called it “cross-dressing.” I corrected him: there is no such thing as “a boy wearing women’s clothes.” If you are a boy, and you wear a skirt, then in that moment, the skirt is simply menswear. Similarly, when I refused to watch a film by saying, *“I’m a girl, I’m not interested,”* he corrected me: *“Whether you’re male or female, you can simply not be interested. That’s a matter of personal preference, not gender.”*

Gradually, I felt freer to “be myself” with him. In broader society, we are bound by countless rules: as students, we must be good pupils; as adults, emotionally stable workers; as friends, people with boundaries; as children, filial sons or daughters. But the biggest and most rigid rule of all is *gender.*

Standards of being a “good woman” are endless. On the macro level: a woman who hasn’t given birth is considered incomplete; a woman who asks for bride price is vain; a stay-at-home mother is unambitious; a female PhD is “undesirable”; a woman who dresses provocatively is deemed immoral. On the micro level: a girl’s phone brand supposedly reveals her temperament; women at the gym should train legs and hips but not upper body; women must wear safety shorts under skirts; using certain words makes a woman “unladylike.”

We may not live by all of these rules, but we see them everywhere. So when a man wants to wear a skirt, he fears being ridiculed as “perverse” or “effeminate.” When a woman wants to lounge on the couch in oversized shorts eating watermelon, she fears being criticized as “sloppy” or “unfeminine.”

In our relationship, much of this fear has dissolved. I sometimes think of my boyfriend as my “best girlfriend,” while he may see me as his “best brother.” It doesn’t matter. The point is: with each other, we no longer need to be “man” or “woman.”

Article URL (optional): <https://mp.weixin.qq.com/s/Jj6nPQmGTJKM-E7iieHstA>

---

Article 10

Public Account Name: Gender studies horizon

Article Title: New trends and characteristics in China's marriage and family structures

Date of Publication:  2023 July 19

Full Article Text:

Editor’s Note
On July 8, 2023, the sub-forum *“New Trends and Characteristics of Marriage and Family Changes in China”* was held at Nankai University as part of the 2023 Annual Conference of the Chinese Sociological Association. The forum focused on new phenomena, trends, and features of marriage and family changes in China’s new era. Participating scholars engaged in keynote speeches, paper exchanges, discussions, and reviews, addressing topics such as *“Marriage and Family Relations in the New Development Stage,”* *“Changes in Family Generational Structures and Intergenerational Relations,”* *“Low Fertility Rates and Family-Friendly Policies,”* and *“Marriage and Family in the Context of Social Structural Transformation.”*

Marriage and Family Relations in the New Development Stage

In this stage, new media platforms and marital status intersect in complex ways. Liu Zixi (Associate Professor, Xiamen University), through fieldwork on nonprofit matchmaking platforms, found that users’ *“social identity positioning”* and *“perceptions of marital compatibility”* are key factors influencing their boundaries of privacy. Ma Kairong (Master’s student, Zhongnan University of Economics and Law), using survey data from 276 cities, concluded that new media usage intensifies marital anxiety among younger generations. Shen Yang (Associate Professor, Shanghai Jiao Tong University) applied grounded theory to show that compared with men, women’s marital and relationship views exhibit greater tendencies toward de-institutionalization; gender differences in perceptions of marriage and risk can lead to mismatched expectations. Liu Junfei (Counselor, Chengdu University of Literature and Science) compared mate preferences among highly educated youth in Chengdu and Taiyuan, finding little significant difference between the two cities.

Marriage forms and family behaviors are increasingly diverse. Xu Qi (Associate Professor, Nanjing University) found that the underlying logic and motivations of family behavior differ significantly from the past. Huang Mingxue (Doctoral student, China Agricultural University) studied the *“dual-family marriage”* phenomenon in Tangcun, Zhejiang, noting that while it fosters more equal spousal relations, it can also trigger crises in intergenerational caregiving and inheritance. Shen Xiaojie (Doctoral student, Peking University), analyzing the *“Chinese College Students’ Marriage and Relationship Survey,”* discovered that second-generation university students express lower marriage intentions than the first generation; moreover, horizontal stratification in higher education produces heterogeneous attitudes toward marriage.

Different parenting models are shaped by diverse factors. Chen Jia (Associate Professor, Shanghai University), from the perspective of grandparental authority, found that grandparents’ psychological control exerts significant effects on both positive and negative dimensions of intergenerational co-parenting. Ma Jiefi (Master’s student, Anhui Jianzhu University) applied survival analysis to show that labor participation significantly delays first marriage and first childbirth, with stronger effects on women.

Changes in Family Generational Structures and Intergenerational Relations

Ideas about interpersonal interaction vary by generation. Cao Ge (Associate Professor, Ningxia University) found that those born in the 1960s display more traditional Chinese cultural values, while those born in the 1980s embody more Western individualist traits. Wan Liangxingzi (Master’s student, Sun Yat-sen University) examined 12 young mothers in confinement centers, analyzing how women from two generations use these institutions’ services and spaces to practice agency in the process of individualization.

Kinship networks closely shape interpersonal networks. Shen Yi (Professor, Nanjing University) contrasted cases to clarify two kinship-network types in human interactions: *“self-interested detachment”* and *“over-embeddedness,”* pointing out the tension between obligation and interest. Xiang Jiangnan (Doctoral student, University of Chinese Academy of Social Sciences) conducted fieldwork on negotiations between parental families aimed at balancing power, analyzing how dual-family marriages influence marital relations and individual autonomy.

Regarding children’s influence on parents: Weng Tangmei (Lecturer, Henan Normal University) found that elderly women who migrate with their children face challenges in adapting to new environments, while elderly men left behind in rural areas often experience loneliness. Zhuo Weijia (Doctoral student, Tsinghua University) showed that in caregiving support, children’s *“parental authority”* is institutionalized by formal management norms; in social support, children help parents manage networks and resocialize, further embedding authority into institutional life.

Regarding parents’ influence on children: Guo Ge (Lecturer, University of Science and Technology Beijing) revealed that some caregivers adopt strategies such as social avoidance, performative parenting, and “dual-faced” childrearing to counter stigma. Wu Xiaolu (Master’s student, Fudan University) studied military fathers and found that they seek balance between professional and paternal roles, with compensatory strategies such as “migratory” parenting, phone calls, or forms of *“absent presence.”*

Low Fertility Rates and Family-Friendly Policies

Fertility intentions are shaped by intergenerational relations, birth order, and family factors. Tian Wenze (Doctoral student, Nankai University), using CFPS 2018 data, found that “dependence on parents” significantly reduces fertility intentions, while “filial piety” significantly increases them. Fang Ying (Associate Professor, Guangzhou University), through interviews with 21 respondents in the Chaoshan region, observed increasingly diverse preferences regarding children’s gender. Qing Shisong (Professor, East China Normal University) found that when husbands consistently support gender equality, wives’ fertility intentions significantly increase.

Fertility concepts are shaped by modernization and structural realities. Wu Yuzhuo (Master’s student, Central University of Finance and Economics) and He Wenyi (Master’s student, Sichuan University) showed, using CFPS 2018 data, that modernized fertility concepts are guided by both workplace characteristics and individual status. Ge Chenlu (Master’s student, Huazhong University of Science and Technology) argued from communitarian theory that reproductive choices emerge as historically and contextually situated balances between independence and dependence.

Family-support policies influence fertility outcomes. Zhang Yang (Lecturer, Renmin University of China) analyzed fertility rebounds in 27 countries, finding that dual-earner–oriented policy packages better support fertility recovery. Lu Chong (Lecturer, Southwestern University of Finance and Economics), using experimental surveys, found that balanced family-support models more positively affect willingness to have a second child than traditional ones. Yu Meng (Assistant Researcher, Nanjing University), applying a policy paradox framework, pointed out contradictions in Korea’s family-support policies.

Marriage and Family in the Context of Social Structural Transformation

Individuals and families are shaped by policies, legal frameworks, and cultural values. Wu Zhen (Associate Researcher, Shanghai University) reviewed the history of French family policy to discuss the persistence of individualization in society. Yuan Xinli (Professor, Lanzhou University of Technology) argued that the *Family Education Promotion Law* advances children’s growth by safeguarding, guiding, and supporting family education. Wang Xiangxian (Professor, Shandong University) stressed that paternal involvement is key to balancing traditional industrial-era family models with newer models, and to advancing the “second half” of the gender revolution in which men enter domestic caregiving.

Individual choices, in turn, shape policies and even culture. Guo Yunwei (Assistant Researcher, Chinese Academy of Social Sciences) showed that Germany, once relying on family-centered childcare, is now expanding public childcare provision. Ji Yingchun (Professor, Shanghai University) examined the matchmaking corner in Shanghai’s People’s Park, finding a hybrid of traditional and modern marital/sexual norms, with distinct gendered boundaries. Kou Kailiang (Doctoral student, Jilin University) found that traditional family-centered values prioritizing household interests are being integrated with developmentalist values emphasizing wealth accumulation, forming a new familialist culture.

Marriage patterns are becoming less stable as social structures evolve, with new residential arrangements emerging. Zhou Han (Assistant Researcher, Jilin Academy of Social Sciences), analyzing census and civil-affairs statistics, found a decline in marriages, rising divorce and remarriage rates, delayed age at first marriage, and later childbirth. Wu Fan (Associate Professor, Guangxi University) reported that among couples living apart, satisfaction with family relationships remains high, with family dynamics and economic conditions being key factors influencing subjective happiness.

Article URL (optional): <https://mp.weixin.qq.com/s/EFLTjA5_rQ4T-WwvjbnTSQ>

---

Article 11

Public Account Name: A green apricot

Article Title: Reflections on physical competition shows: do women need to be strong to earn respect?

Date of Publication: 2023 December 04

Full Article Text:

This year, the Korean variety show *Siren: Survive the Island* became a huge hit. The program invited 24 women from different professions—stunt performers, soldiers, firefighters, police officers, security guards, and athletes—almost all of which are stereotypically seen as “male occupations.” In China, a new fitness competition show, *I Can 47,* has also featured women. It invited 47 athletes and fitness bloggers from different fields, with men and women required to form mixed teams. Most competitions were not gender-segregated.

In these physical competitions, I saw women who looked very different from the East Asian aesthetic ideal of being pale, thin, and delicate. These shows created an opportunity to break gender stereotypes and to demonstrate that women can rival men not only in physical strength but also in mental toughness.

Although I personally have almost no experience in combative sports, watching these women’s performances was exhilarating. Their fighting spirit and ambition are qualities we rarely see in female portrayals in mainstream media. Strong women are never absent in real life—but the camera seldom points at them. Still, as inspired as I felt, I also noticed a contradiction: should women’s strength be measured by showing they can be “as strong as men,” adopting the same displays of physicality? Or should there be differentiated narratives and evaluative standards for female strength?

These shows also expose the stark reality of the *“strongest wins”* logic. While some exaggeration comes from editing, the competitiveness is undeniably real. In *Siren: Survive the Island,* the women fought fiercely, turning the contest into a kind of “war” among women from different professions. Similarly, *I Can 47* emphasized that the competition was *only for the strong.*

After an opening climbing challenge, the top six competitors (all men) automatically became team captains. Given the emphasis on raw strength, many did not want to team up with women, whom they assumed were weaker, even if some female athletes specialized in strength. Women were sometimes dismissed during the team selection process. Male competitors whose expertise lay in technique rather than raw power were also disparagingly called “thin dogs” and undervalued. In the end, the team considered the strongest had only one woman—the lowest female representation among all six teams.

Interestingly, physical strength was not the only factor in choosing captains. The sole female captain, Zhang Yuhan, gained her role because the male competitor who ranked second voluntarily declined the position. He felt that while he had raw strength, he lacked the leadership qualities required of a captain, and so suggested the role be passed to the seventh-place finisher.

The shows were not devoid of warmth and humanity. For instance, in one challenge requiring teams to build a suspension bridge and transport supplies, the competition ended with everyone leaping off the cliffside bridge onto padded mats. Many contestants revealed their fear of heights, showing that even champions have vulnerabilities. In those moments, competitors set aside rivalry, encouraged each other, and celebrated the collective achievement.

Yet the dominant tone remained one of *winner-takes-all.* Strength was defined not by perseverance or willpower, but by results. Many female contestants deliberately erased their gender identity, declaring: *“I don’t see the women here as women, nor the men as men,”* or *“This show is genderless, I just give it my all.”* This was a way of rejecting the gaze, framing, and constraints imposed on women.

Still, some contest designs ignored differences in male and female physiology, as well as the unequal gender distribution across teams, leaving female-heavy groups at a disadvantage. Audience commentary also revealed a bias toward male strength. The only female captain drew enormous attention but also the most controversy. Some viewers questioned her competence, saying that while Zhang Yuhan was friendly, she lacked decision-making ability and leadership skills, and should have been replaced.

These shows do celebrate exceptional women who surpass ordinary physical limits. Yet their victories often seem disconnected from *female strength* as such. For viewers like us, who may struggle just to jog three kilometers, what we see on screen is less about personal inspiration than about a cultural narrative: in modern high-speed society, *“only the strongest deserve recognition.”* This logic has long been used to marginalize women—dismissing or erasing them because they are deemed “not strong enough.” Often, however, women’s “weakness” is not about lack of willpower, but about structural conditions and life circumstances.

I certainly hope to see more shows spotlighting strong women, inspiring others to pursue paths traditionally coded as male. But what I do not wish to see is a world—real or constructed by television—where there is only one kind of victory: victory defined by male standards of strength.

Article URL (optional): <https://mp.weixin.qq.com/s/a96glycJ2wzgoPH18CeoTA>

---

Article 12

Public Account Name: Gender studies horizon

Article Title: Changing patterns of childbearing intentions among Chinese women of reproductive age

Date of Publication:  2023 October 20

A nation’s fertility potential depends on both the size of its childbearing-age population and their fertility intentions. The number of women of reproductive age is determined by past birth cohorts. According to China’s existing population data, the size of women—especially those in their prime reproductive years—is shrinking, a trend that cannot be reversed in the short term. As a result, fertility intentions have become a decisive factor in shaping future fertility levels and the scale of newborn populations.

Fertility intention refers to people’s subjective aspirations regarding their fertility goals or behaviors—whether to have more or fewer children, to have them earlier or later, or to prefer boys or girls. Among these, the intended number of children is the most central indicator. Commonly used measures include the *ideal number of children,* the *intended number of children,* and the *fertility plan.* The ideal number reflects the socially normative family size and may change alongside societal development. The intended number refers to how many children an individual or couple hopes or intends to have. Fertility plans capture more concrete expectations, such as whether one plans to have (or have additional) children, and the anticipated timing. Research shows that the *ideal number of children* is the most stable measure; fertility plans are more prone to short-term change, but concrete plans regarding number and timing are more likely to translate into actual behavior. Compared with the ideal number, the intended number has greater policy relevance and practical value.

Fertility intention is shaped by both macro-level and micro-level factors. At the macro level, national population policies, economic development, traditional culture, modes of production, social security systems, and employment conditions exert significant influence, often affecting fertility intentions across regions or even entire nations. At the family level, the cost of childrearing and the contributions children make to family life play important roles. At the individual level, factors such as age, gender, and educational attainment both underpin and interact with family- and society-level influences.

Recent trends in fertility intentions among Chinese women of childbearing age reveal several features:

First, overall fertility intentions remain low. The 1985 National Fertility Survey and the 1990 China Women’s Social Status Survey found that Chinese women’s ideal number of children was 2.40 and 2.23, respectively. A 2002 nationwide urban-rural fertility intention survey showed that, absent policy restrictions, the ideal number had fallen to 2.04. Subsequent national fertility surveys have consistently reported average ideals below 2.

Second, fertility intentions are on a downward trajectory. National sample surveys conducted in 2017, 2019, and 2021 by the National Health Commission found that women’s average ideal number of children declined from 1.96 (2017) to 1.92 (2019) and 1.84 (2021). Over the same period, the intended number of children dropped from 1.76 to 1.73 and then to 1.64.

Third, the share of low-intention groups has increased. Across these surveys, over 70% of women reported an ideal of two children, but the decline in averages reflects the rising proportion of those with lower fertility ideals. Low fertility intentions are particularly concentrated among young people and residents of first-tier cities, where high living costs, intense pressures, and greater opportunity costs of childbearing dampen aspirations. Addressing the low fertility intentions of younger cohorts is crucial for boosting overall fertility willingness.

At the individual level, fertility intentions exhibit relative stability, but are also shaped by formative experiences in family education, schooling, early employment, and marriage. Personal reproductive experiences may also shift fertility aspirations. The 2021 national tracking survey (following the 2019 survey) found that 74.5% of women reported consistent fertility ideals across both waves, 11.1% reported an increase, and 14.3% reported a decrease. The ideal of two children proved the most stable.

According to the framework proposed by U.S. demographer John Bongaarts, the total fertility rate (TFR) is influenced not only by fertility intentions, but also by three positive factors that push actual fertility above intended levels, and three negative factors that depress it below intended levels. Positive factors include unintended births, replacement of deceased children, and sex preference. Negative factors include delayed childbearing, involuntary infertility, and competing life pressures. Among low-intention groups, however, the positive factors of replacement and sex preference exert limited influence. While lifetime childlessness remains low in China, the growing spread of the attitude that “it doesn’t matter whether I have children” among younger generations cannot be ignored. Persistently low first-birth rates would inevitably dampen the likelihood of second and third births.

Understanding fertility intentions and their evolution is thus key to predicting future fertility trends. To achieve a moderate fertility level, it is first necessary to track and interpret shifts in intentions. As socioeconomic development increasingly shapes fertility behavior, proactive family-supportive policies are essential for creating a favorable environment. Whether through enhancing maternal and child health services, reducing the costs of childbearing and childrearing, or expanding accessible childcare services, such measures all aim to ease “back-end concerns.” Ultimately, fertility intentions signal whether people *want* to have children even before they act. Only by first addressing the problem of “not wanting children” can subsequent policy measures be truly effective.

Article URL (optional): <https://mp.weixin.qq.com/s/Vg4cAF9AKs5glhVmOqP9DA>

---

Article 13

Public Account Name: [GenderDream](javascript:void(0);)

Article Title: After menstruation “left,” Xiao fu discovered a new way of relating to it

Date of Publication: 2023 June 09

Full Article Text:

From the end of December 2022 until March 2023, after recovering from COVID-19, Xiaofu experienced a three-month delay in her menstrual cycle. The sudden change overwhelmed her with stress and anxiety, prompting her to begin trying to “talk” with her own body. When asked how she viewed menstruation, Xiaofu answered: *“Menstruation is my companion.”*

Before the “less-than-ideal” experience

For Xiaofu, menstruation used to be a *“like-minded companion.”* From high school through university, she was never far from friends troubled by menstrual issues or related health conditions. When recalling her friends’ experiences, she instinctively said: *“It just felt so hard, so painful.”* This hardship included a friend who, due to polycystic ovary syndrome (PCOS), shuttled between school and the campus hospital, and dormitory life filled with heating pads, ibuprofen, and brown sugar for roommates suffering from cramps.

For Xiaofu, however, she always experienced these things from a third-person perspective. Her friends’ stories, or the menstrual health tips occasionally appearing in WeChat chats, felt both near and far. Subconsciously, she never really considered herself part of the *“troubled group.”* The brief discomfort of cramps was something she associated only with middle school. But under the triple pressures of academics, family, and the pandemic, her cycle delays grew too significant to ignore—making her feel betrayed by what had once been a perfectly synchronized “partner.”

Betrayal and anxiety

The absence of her “companion” carried the hidden fear of pregnancy, which became a direct source of anxiety. *“Pregnancy would ruin all my plans. I can’t get pregnant.”* This became a persistent, intuitive fear. Even without dramatic physical changes, the potential disruption of her academic and family routines generated enormous psychological pressure. *“That first month after COVID recovery, I completely collapsed. I felt mentally destroyed.”*

With her partner’s encouragement, Xiaofu went to the hospital. She was diagnosed with hyperandrogenism and PCOS. When the doctor asked, *“Has your body hair increased?”* Xiaofu suddenly noticed that the smooth arms she once took pride in had indeed become hairy. Her body was changing. The acne breaking out on her chin and forehead, the frequent mouth ulcers, the weight gain she once attributed to eating habits—all of these now seemed connected.

When she saw the prescription—*“Begin taking Yasmin (a short-term contraceptive) on the first day of your next period”*—she thought: *“But this makes me feel depressed.”* (Editor’s note: medication should always be taken under professional guidance.)

The doctor’s only reply was: *“There’s nothing else we can do.”* At the pharmacy, Xiaofu knew she had chosen to resolve her “menstrual problem” at the expense of her emotional well-being. It felt like a message from her “companion”—a signal that things were moving in an unwanted direction.

Signals and self-doubt

The B-ultrasound confirming she was not pregnant did not bring relief but rather deep frustration. When anxiety itself seemed to be the only “cause,” Xiaofu fell into a spiral of self-denial: *“Why am I so anxious that my body has gone abnormal?”*

Not menstruating became a curse, haunting her daily moments—sitting in a chair, scrolling on her phone, only to be suddenly seized by agitation. She blamed her cycle’s uncontrollability and often felt irritable.

Her partner’s sharing of an article, *“Having PCOS is a kind of gift,”* eased her mood. She didn’t read carefully, but it made her wonder: perhaps it was her environment that had taught her to fear and reject her body. From another perspective, maybe it wasn’t such a problem.

A brief conversation with her father reinforced this idea. Their relationship had always been distant, so she expected little when she mentioned her diagnosis. But the next time they met, he asked while pouring tea: *“Have you taken your medicine?”* That single question surprised her. For the first time, she realized menstruation was not only a signal to herself but also a signal to others.

Redefining the “companion”

*“Menstruation has become my way of resisting, a way of venting.”* To parents and to herself, she often repeated: *“I’m under too much pressure already. Please don’t add more.”* These repeated declarations lowered others’ expectations and gave Xiaofu space to renegotiate her relationships and her own self-expectations. She began to sort through pressures, responsibilities, and ties—choosing what to prioritize.

Academic and life stresses that once felt overwhelming now seemed trivial compared to her body’s warning signs. By contrast, her parents’ care and her partner’s support became more tangible and meaningful. She kept visiting her parents as before, but instead of exhaustion, she now felt the importance of spending limited time with them. *“Knowing what really matters, and what I can’t do,”* became her new principle.

Acceptance and renewal

In early April, when her cycle “normalized” again, Xiaofu breathed a sigh of relief. This time, taking Yasmin did not trigger the mood swings she had feared. At the same time, she was preparing for an upcoming trip with her partner to Thailand. *“Because of the pandemic, I missed my graduation trip. I regretted it for so long, felt like life had been wasted. But now I can finally make it up.”* Her voice was filled with joy and anticipation.

In hindsight, Xiaofu feels this unusual menstrual experience gave her more courage and determination to change. The illness itself was not as negative as she had once thought. Reflecting on her relationship with menstruation, she now speaks without disappointment or self-blame: *“I think we just unconsciously entered a new stage. Facing the signals head-on allows me to adjust my mindset, rather than blaming the problem for suddenly arriving. Things are simply different now.”*

Article URL (optional): <https://mp.weixin.qq.com/s/zbFruje2LKmLCNe4Bzq2eg>

Article 14

Public Account Name:  [GenderDream](javascript:void(0);)

Article Title: How can asexual perspectives inform our understanding of sex education?

Date of Publication: 2023 October 20

Full Article Text:

My first formal sex education took place in the second year of middle school.
When our biology class reached the unit on human reproduction, students were then split—by assigned sex—into separate “sex education” sessions. I was placed in the girls’ class, where I soon realized the content on menstruation and related topics could have been covered in biology; for some inexplicable reason, these topics were outside the exam syllabus and off-limits to roughly half the students.

A considerable portion of that class was spent dramatizing how “horrifying” abortion surgery is—so dull I could hardly stop yawning.

Only in high school, after encountering feminist literature and re-educating myself, did I begin to grasp what sound sex education should look like—and how much that earlier class had missed: treating reproduction as the *sole* purpose of sex; omitting contraception; refusing to address sex’s social meanings and sexual pleasure; enforcing cis-heteronormativity; and more.

Back then, I also didn’t feel that sex education beyond menstruation had anything to do with me. Even in high school, I treated “sex” as an elective track under gender justice—useful, like phonetic symbols for learning an alphabetic language, but not strictly necessary.

When I first entered university, I encountered the concept of asexuality / lack of sexual attraction and gradually identified as a person who experiences little or no sexual attraction. Yet unlike many people elsewhere online, I wasn’t outraged that asexuality was missing from my sex education.

Two reasons may explain this: In the society where I grew up, heterosexuality—the only recognized norm—was not even regarded as an “orientation.” Babies were “a free add-on with your phone plan,” as the joke goes. Only non-heterosexuality was linked explicitly to “sex,” and therefore treated as purely sexual. In an environment of sexual repression, queer communities—as well as anything sex-related—were pushed into the shadows. No one mentioning asexuality felt utterly normal. The invisibility of asexuality did not impact me much as a teenager. Adults insisted sex was not for children to know or do, and peers who knew anything wouldn’t say so loudly. My lack of interest in sexual talk was merely one of my many “quirks,” and not a conspicuous one.

When I tell friends from other places about this, some express surprise and recall how they once pretended to “like” someone (automatically conflated with *sexual* attraction) to fit in, or how they felt overwhelmed by ubiquitous sexualized chatter. I am not defending sexual repression or queer erasure. I want to highlight a distinct situation that some asexual people face compared with other queer groups.

Of course, after I reached adulthood and discussed future plans with my family, I clearly felt sexual discipline tightening around me. On the one hand, no one should be subject to such regulation. On the other hand, my resistance to intimate relationships and marriage (not all asexual people feel this way) made my family even more anxious—since they assume everyone must inevitably be sexually active, and anything sexual outside stable relationships or marriage is dangerous. When attempts to explain failed, I truly recognized how asexual visibility, sex education, and sexual liberation are interdependent.

Note: We are advocating renaming “无性恋 (wuxinglian)” to “无性吸引 (lack of sexual attraction)” to emphasize that *asexual* concerns the absence or rarity of sexual attraction, and to avoid conflating sexual attraction with romantic attraction. See our public account’s custom menu for the rationale behind this change.

It’s Not Enough to “Add One More Option”

A classic complaint about queer visibility concerns the multiplication of gender options. Transphobes say choosing from “ten+ options” is impossibly hard. First, we regret such narrowness. Second, we must point out that simply adding options often amounts to laziness and irresponsibility.

Leaving aside the odd decision to separate “trans women/men” from “cis women/men,” in most cases, option-setters merely want to appear progressive without improving queer lives. This exhaustive, box-checking approach is tokenism—a parallel to inviting one or two people from a marginalized group to “make up the numbers” while denying them real voice.

The widely praised British series *Sex Education* falls into tokenism with its asexual character. In season two, the character Florence feels out of place at a school where everyone talks about and has sex, and she worries that her lack of interest in sex means she is “broken.” She seeks out the sex therapist Jean, who tells her: *“Sex doesn’t make us whole. How can you ever be broken?”*

This seemingly positive portrayal is problematic: Florence appears for under five minutes, and her presence is irrelevant to the plot—there to showcase the show’s “inclusivity.” The show reduces asexuality to “not wanting sex,” ignoring the wider spectrum of experiences. For many viewers, this is their only exposure to asexuality—thus misleading. Despite Jean’s affirmation, the series is saturated with “aphobia”: nearly every character who cannot enjoy sex is framed as troubled or “ill,” and ultimately “fixed” so they can enjoy sex. Sex and romance are routinely conflated. The protagonist Otis could have been a nuanced asexual or gray-asexual figure—highly sex-averse in a hypersexual environment—but his mother and friends pathologize him, chalking it up to childhood trauma. By the end of season one, he miraculously becomes “normal” in front of the person he likes.

The root problem is the show’s refusal to let asexuals challenge the sex-centric norm. Flattened as she is, Florence cannot pose crucial questions: What if “liking someone” is unrelated to sexual desire or sexual attraction? What if sexual desire, sexual attraction, and sexual behavior are not necessarily linked? Just as the term “transgender” destabilizes the assumed norm of “cisgender,” the emergence of asexuality challenges the hegemony of “heterosexuality/homosexuality/bisexuality,” compelling us to reconsider definitions of sex, and the separation of sexual and romantic attraction. Therefore, adding “asexual” to a drop-down or placing an asexual character on screen is progress—but we still have a long way to go.

Hard-Won Sex Education and “Asexual” Children

As argued above, robust sex education benefits everyone, including those with little/no sexual or romantic attraction. Yet, even today, most people learn from the internet or academic books, both requiring resources and the ability to assess accuracy and recency. Schools should be the most systematic, convenient, and intensive setting for learning, but sex education remains hesitant and easily derailed by parental or public opposition.

Beyond obvious sexual control and repression, a key reason is society’s denial of childhood sexuality and minors’ subjective rights. A common dismissal faced by asexual or aromantic people is: *“You’ll be interested when you grow up.”* This presumes sex and romance are adult privileges and casts asexuals and aromantics as childlike—incapable and thus invalidating their feelings.

In reality, leaving aside that asexual people may engage in sex, longstanding research shows that many human children are not entirely insulated from sexuality. This article will not delve into the complexities of prepubescent sexual behavior, nor equate it with adult sexuality. The point is: Using “you’re too young” to negate asexual feelings is baseless. Empowerment for asexuals parallels minors’ struggles against denial of agency. Denying childhood sexuality is a tool of sexual governance—splitting human sexual development (a continuum) under a veneer of science, rendering sex education taboo and increasing minors’ risk of harm. The forced split between children’s and adults’ sexuality fuels the myth that LGBTQ+ people “corrupt” children—as if none of us were once queer kids ourselves.

Before the eighteenth century, talk of children’s sexuality was rare; “child” was not widely recognized as a distinct human category, and the sharp child–adult divide was a modern social construction. With the rise of sexology and psychiatry, children, women, people of color, and other marginalized groups were placed under scrutiny (e.g., hysteria; children’s masturbation deemed gravely harmful). These ideas spread across Western Europe in the nineteenth century, where children’s sexuality was seen as a societal disease.

Freud later argued, based on numerous cases, that infant and child sexuality is normal and harmless, and that repression and mishandling cause harm. Why, then, does our society remain evasive? One reason is the misuse of Freud to defend those who abuse children. The feminist movements of the 1970s in Europe and the U.S. made major contributions to anti-violence, especially resisting the “Lolita” myth that sexualizes victims. Yet to avoid giving ammunition to abusers, some feminists denied any relation between children and sexuality altogether.

Sociologist David Finkelhor, for example, argued children neither understand sex nor can consent to it when facing adults, therefore can never consent under any circumstances. While we recognize vast power asymmetries between adults and children, portraying children as wholly “ignorant of sex” is harmful. Each subject’s relation to power is unique and shifting; sex is only one factor among many. Treating “sexual immaturity” as *the* cause of adult–child power gaps enshrines sexual hegemony, obscures non-sexual abuses of power, and hides how society disempowers children—e.g., withholding needed sex knowledge, policing and pathologizing exploration. Consent capacity is not acquired by a birthday. From before birth onward, cis-heteronormative messaging saturates children’s worlds; taboos ironically transmit plenty of sexual norms. Erasing children’s sexuality does not prevent abuse; it can deepen victims’ helplessness and shame, even when they possessed *some* awareness that still falls short of consent.

Society’s preference for the “perfect victim”—the innocent, desireless child—makes prosecutions appear easier. But this image usually applies to middle-class majority children; heterosexuality and majority race are invisible norms, whereas queer and minority children are hypervisible and judged. For people of color, the poor, sex workers, queer folks—indeed anyone outside dominant sexual norms—even as minors, society presumes sexual desire or activity and then blames them, by a bandit logic of “having desire = wanting violation.” Even “perfect victims” often lose due to invented stigma, rarely benefiting from the split between desire and rights.

Current sex education is largely regulatory: focusing on preventing assault and unintended pregnancy while treating desire and pleasure as taboos for minors and privileges for adults; binding sex, romance, intimacy, and marriage together; and omitting sex and intimacy education as parts of life. Even the “prevention” content largely ignores trans people, non-heterosexuals, and the stark differences created by urban–rural and class divides.

Only when we can speak about sex openly—neither romanticizing it nor demonizing it—can we thoroughly rethink and unpack what “sex” means, allow asexual people to breathe freely, and leave abuses of power nowhere to hide.

Article URL (optional): <https://mp.weixin.qq.com/s/8iQU7fDHsqgCxr2ISDCwNg>

---

Article 15

Public Account Name: [GenderDream](javascript:void(0);)

Article Title: Expectation, limitation, and identification: why do children align with traditional gender roles?

Date of Publication: 2023 December 06

Full Article Text:

The process by which children gradually form self-awareness is known as the socialization of children. Social learning theory holds that, through socialization agents, children learn social knowledge and norms and ultimately become “members of society.”

Gender is socially constructed, and the formation of gender roles is one of the key manifestations of socialization. This article focuses on how children’s understanding of gender roles develops; it discusses how various socialization agents that shape children embody contemporary society’s gender expectations and present masculinities and femininities within the traditional gender order.

From a process perspective, socialization does not act on individuals directly; it is conveyed through intermediary mechanisms. In sociology, these are termed socialization agents, that is, “groups or contexts within which significant socialization processes take place.” Concretely, the chief agents include family, school, and media, which jointly influence children’s gender-role socialization.

The Socializing Family

Early Gender Differentiation and Childrearing

The family is the smallest unit of society. In children’s gender socialization, the family functions as the earliest socialization agent; parents’ childrearing styles and behaviors play a crucial role in shaping children’s gender consciousness. “Parents are not only caregivers but also the earliest shapers of a child’s psychology and behavior. Parental attitudes toward gender-role socialization exert a non-negligible influence on children’s development.”

First, parenting style affects a child’s gender identity. Because gender beliefs differ, parenting styles do as well. A rigid, monolithic gender belief appears as a gender schema, i.e., “a fixed set of views and expectations about men and women.” As a cognitive structure for information processing, gender schema is a direct projection of gender stereotypes; parents with traditional schemas tend to raise children through traditional stereotypical expectations.

Such schemas permeate everyday family life—from color preferences and clothing to toys: girls “should” wear pink dresses and hold dolls; boys “should” wear blue shorts and play with robots and toy cars. In the popular parenting reality show *Where Are We Going, Dad?*, actor Hu Jun often used a stern tone with his son, urging him to “be manly,” while stating in interviews that he doted more on his elder daughter and was stricter with his son. This sex-differentiated pedagogy and the “little man” expectation illustrate how schema-driven individuals hold stereotyped understandings of masculinity and femininity. Children internalize and recode such schemas, gradually establishing stereotyped self-scrutiny and gender identity that converge on traditional gendered dispositions.

Second, children model their parents’ genders. Psychoanalyst Sigmund Freud argued that infants lack gender differentiation; gender awareness sprouts through interactions with parents. He attributed a key manifestation of gender differentiation at ages 2–6 to the Oedipus complex (boys) and Electra complex (girls), proposing that children model the behaviors of the opposite-sex parent. This biologically driven view of gender roles remains contested; many scholars instead emphasize that gendered psychology is the product of social learning, i.e., “childhood, unlike infancy, is a social product, not a purely biological domain.”

With evolving social concepts, the family’s role in children’s gender-role development has drawn broad attention. In 2017, the BBC documentary “No More Boys and Girls?” (on gendered toys) probed parental gender stereotyping in childrearing. In 2019, Barbie-maker Mattel launched a gender-neutral doll, stating that the dolls are not limited by gender identity and that all children should grow up in inclusive environments where they can freely express themselves. Gender education within the family is thus no longer a purely private matter; it is entering the public arena, and diverse gender expressions are increasingly accepted and encouraged.

The Socializing School

“Authoritative” Models and Expectations of Gender Roles

According to Lawrence Kohlberg’s cognitive-developmental theory, children’s gender-role development tracks their cognitive growth and proceeds through three stages: basic gender identity (ages 2–3), gender stability (4–5), and gender constancy (5–6). Most children at these ages are in kindergarten, making it a critical period for laying the basic framework of gender-role cognition.

To avoid gender bias and promote equality, proposals for “gender-neutral education in kindergartens” have emerged. In 2010, with support from Sweden’s Ministry of Education, the Egalia preschool in Stockholm adopted a gender-neutral approach: no explicitly gendered objects in the environment; teachers blurred gender boundaries in communication, used gender-neutral terms, and avoided “he/she,” aiming to prevent fixed gender impressions.

Debates between “gendered education” and “gender-neutral education” remain lively. As a site of authority, the school provides concentrated learning and formal curricula; its gender socialization often lands on establishing “standard” role models, primarily through teachers’ expectations and curriculum content.

On teachers: “Teachers are the child’s first authority beyond the family.” Children are impressed by teachers’ authority, deem their words and behaviors highly credible, and actively respond to gendered expectations. With great power comes great responsibility: teachers’ gender sensitivity is vital in guiding gender roles. Meanwhile, teacher workforces have grown more gender-skewed. The OECD’s 2017 report “The Gender Imbalance in the Teaching Profession” noted that women made up 68% of teachers (2014).

On content: textbook bias is widely criticized. Textbooks, as foundational teaching materials, should be rigorous and neutral; yet gender bias persists. An analysis by Wang Songlin (2010) of post-1999 junior-high English textbooks found marked differences in portrayed occupations: women were more often in “affective” roles (teachers, shop assistants), while men appeared in innovative or physically demanding roles (IT staff, drivers, doctors). Such role models reflect strong gender bias.

The Socializing Media

Visual Depiction and Repetition of Gender Roles

As a key channel for perceiving the world, media promotes socialization. Since the 1970s, communication scholars have probed the media–socialization nexus. Under the notion that “the medium is the environment,” the media ecology school (Neil Postman) argues that media environments closely correlate with socialization. In *The Disappearance of Childhood*, Postman explored television culture’s impact on children, suggesting that TV exposes them prematurely to adult information, thereby eroding childhood. Psychologist David Elkind likewise argued that children immersed in ubiquitous media are robbed of childhood.

This “disappearing childhood” thesis remains relevant in the digital era. With economic growth and technological infrastructure, younger and younger children use smartphones. The Blue Book on Youth: Report on Internet Use by Chinese Minors (2020)—issued by the Institute of Journalism and Communication at CASS, the School of Journalism and Communication at UCASS, and Social Sciences Academic Press—showed that 78% first accessed the internet at age 10 or younger, concentrated between 6–10, summarized as: *“the later one is born, the earlier one goes online.”*

Amid today’s torrent of digital video, low barriers to audiovisual tech extend children’s cognitive reach, making digital media an increasingly important socialization agent. Media influences gender-role socialization chiefly by presenting gendered images. Social learning theory posits that children model and internalize observed roles; media portrayals become ready-made templates. The danger is that content lacking gender awareness harms healthy gender-role development.

On the one hand, media gender portrayals tend to be singular and flat. In the animated series Pleasant Goat and Big Big Wolf, for instance, Feiyangyang (male) is dark-skinned, wears blue, muscular, and into fitness; Meiyangyang (female) is fair-skinned, wears pink, cares about appearance, and likes crafting. Main characters are rendered through traditional femininity/masculinity—simplistic and heavily symbolized.

On the other hand, some children’s animations drift toward adult themes. Take Bond Dream of Yeluo Li (targeting girls aged 7–14): the female lead, a fifth grader, transforms into a sexualized adult body; female interactions are rife with malicious “catfights,” etc. In response to such adult-oriented content, the Shijiazhuang Municipal Bureau of Culture, Radio, TV and Tourism ordered the show removed for rectification. A *Rule of Law Daily* commentary noted the imitative effects on child viewers: “After watching, my 9-year-old daughter complained her legs weren’t long enough and clamored for 8-cm heels.” Accordingly, media producers should bear social responsibility, calibrating content to children’s cognitive levels and perceptual scope to create more precise, appropriate works.

Article URL (optional): <https://mp.weixin.qq.com/s/7583V-GWVc2DEkZrpIWa5g>

---

Article 16

Public Account Name: A green apricot

Article Title: Watching a childhood video of myself running naked, I felt deep shame...

Date of Publication: 2023 July 12

Full Article Text:

1.

Not long ago, while flipping through years-old family photos, a video from over a decade ago shattered my warm memories of childhood and left me deeply unsettled. It was shot in summer, on a crowded beach. There I was—about four or five—at the center of the frame, running and cheering as the waves rolled in.

What made my blood run hot was this: I was completely naked. I had no memory of it at all. If not for the footage, I would never have believed it happened. There was no doubt about who filmed it—my parents, the very people now showing me these images. We seemed to inhabit two different worlds: they watched the video with fondness and nostalgia.

The next clip shocked me even more. I was lying nude on the wet sand, covering my abdomen with it. My parents did not stop me; instead, a jarringly playful voice came through: “Mommy’s playing with you…” A wave of humiliation surged through me. I wanted only to fast-forward—or to leave. Fortunately, something came up at home and my parents had to turn to chores and work. I slipped away and avoided the awkwardness that might have followed.

2.
That video reminded me of something I saw a month earlier while hiking a seaside boardwalk: on the same kind of beach, in the middle of a crowd, a boy of about five or six stood completely naked, facing the ocean, plainly visible even from a distance. The adults nearby seemed to be his parents.

I looked away. My mood soured instantly. I realized I’m not alone in feeling this way. Perhaps boys do not mind at the time—but that doesn’t mean they won’t feel shame when they remember it later. Years on, such a memory may surface and churn their insides. That reaction is not the same as recalling an ordinary childhood embarrassment.

Do little boys really want to be exposed like that in public? Do they understand what removing their underwear means? Are they acting out of free will—or simply trying to be “good children”? They won’t be pondering such questions at that age. But once grown, the facts cannot be undone. Even years later, they may feel the pain of having had their privacy displayed when they were too young to know better—and perhaps resentment that their parents failed to protect that privacy.

When parents dismiss boys’ privacy, they pass on a harmful habit, lowering boys’ respect for and awareness of protecting their own privacy. Gradually and imperceptibly, boys may come to disregard the importance and inviolability of privacy—their own and others’.

3.
And so some boys, in play or horseplay, fixate on each other’s crotch. Last week on the subway, I witnessed something I can’t forget. This was in a prosperous first-tier city, yet things were no better. While I was waiting for the train, a primary schooler ran past me to a young woman—his older sister, perhaps—complaining and gesturing:
“He came up and grabbed his—”

She replied, “You can play, but…” I didn’t catch the rest. It hardly fazed me; I’d seen this often in grade school. On board, though, the scene turned cruder. I stood facing two brothers and an elder. Soon I heard one in a childish voice call the other “pervert.” Looking over, I saw the older boy place his hand on the younger one’s crotch. When the younger stood and pushed his hand away, the older poked from behind. The younger retaliated in kind; now the older was the one shouting “pervert.”

They went on like this while their guardian looked on, seemingly used to it and unable—or unwilling—to intervene. Eventually, perhaps noticing my disapproving look, they stopped. But will anything change? Will the adults tell them not to do this again?

4.
We often insist that girls’ bodies must be carefully protected, their privacy never exposed.
The same should hold for boys. Unfortunately, many people seem to think boys’ privacy matters less. Some violate it in the name of affection; others allow boys to treat touching one another’s private parts as a joke.

How do boys feel? Some blush with embarrassment yet stay obedient and quiet. Others show no reaction—perhaps because they’re less sensitive—not because they will forget. Many do not yet understand their feelings or their bodies, nor do they grasp what they are doing. But such childhood memories don’t vanish; they resurface with age. For most boys, I suspect, these will be memories they’d rather not revisit—memories tinged with shame.

Adults often fail to see this. Fertility worship, the objectification of boys’ privacy, sexualized “jokes” among boys—such norms are common in some places and can cast long shadows. For the sake of the next generation of boys, it’s time to change. I hope that in a few years, when those born in the late ’90s and 2000s become parents, boys will receive proper sex education and their privacy will be fully protected.

Article URL (optional): <https://mp.weixin.qq.com/s/khSEZ8CQbwJfs11s2N922Q>

---

Article 17

Public Account Name: Gender studies horizon

Article Title: The internet as a new frontier for women's development

Date of Publication:  2023 July 27

Full Article Text:

An open, inclusive internet empowers women; highly social, interaction-loving women in turn help build the internet. Over the past decade, China’s internet has surged ahead. Women’s participation, engagement, and contribution have risen year by year—women now constitute an indispensable “half the sky” in China’s digital development.

With the spread of mobile internet, the rise of social media, booming e-commerce, the platform economy, and widespread AI applications—alongside the constant emergence of online influencers—the past ten years have been a golden era for China’s internet.

Data released at the 2023 World Internet Conference Blue Book press event show that from December 2012 to June 2023, the number of Chinese internet users grew from 564 million to 1.079 billion, and internet penetration rose from 42.1% to 76.4%, forming the world’s largest and most vibrant digital society. From 2012 to 2022, China’s digital economy expanded from 11 trillion yuan to 50.2 trillion yuan, leading globally in areas such as internet applications, user scale, and AI development. Chinese women have ridden this digital wave with resolve and momentum.

Women coders: “hidden gems”

On International Women’s Day 2023, China Daily’s bilingual animation “Chinese Women’s Internet Journey” noted that women account for roughly 55% of internet entrepreneurs in China. Notably, more women are appearing in roles traditionally viewed as male-dominated—technology and R&D positions. The highest female representation is in data science, testing, and communications technology. In 2021, women comprised 19.5% of internet/digital technology R&D roles, up 1.6 percentage points year-on-year.

According to the *2022 Female Programmers Insight Report* by Daily Interactive, women make up 22.96% of programmers, mostly aged 18–34. Over 80% fall into mid-to-high consumption tiers. The report portrays female programmers as diligent, financially independent, committed to continuous learning—“hidden gems” who love coding and life alike. A joint report by Tencent Classroom and Liepin found that from 2018 to 2020, the number of female programmers grew by nearly 70%. Women coders rate continuous learning as the most critical workplace skill, spend 1.5× what men spend on learning, and devote more time to it.

One emblematic story is that of Sun Ling, a post-90s woman from rural Loudi, Hunan, who went from a Shenzhen assembly line worker to a highly paid U.S. software engineer in a decade—rising from a 2,300-yuan monthly wage to 1.8 million yuan annually. Forced twice to drop out of school due to poverty, she worked in factories, then paid for coding courses (Java, HTML, etc.) via part-time jobs and credit, completed training, entered an office role, studied English, earned tertiary credentials, and job-hopped her way into better positions. In 2017 she pursued a computer science master’s in the U.S. with internship opportunities, overcame financial and language hurdles, and ultimately became an L4 software engineer at Google. The internet was her toolbox and launchpad at every step.

Another “viral” example is Huang Xuemei of Xiamen—a marathoner and data engineer—who ran 2:32 at the 2023 Beijing Marathon, placing 7th among international women and 3rd domestically (the runners ahead were all professionals). “Work and marathoning are connected,” she says—cleaning data, leading projects, pushing to win. Today’s women engineers write code and live richly: some love fitness, others personal finance, others dote on pets. Lagou’s *2023 Programmers Insight Report* shows women accounted for 26% of new tech-talent registrations in the first three quarters of 2023, a rising share in China’s tech innovation. Women show strong potential in AI and big data R&D.

Du Lan—President of the Guangdong AI Industry Association and Executive Director at Pearl River Investment, formerly an SVP at iFLYTEK—argues that women will be primary beneficiaries of digital technologies. “Ninety percent of AI voices are female,” she notes, because female voices are often perceived as warmer and more relatable. She adds that internet thinking maps naturally onto “female thinking”: valuing emotional experience, multi-centeredness, and sharing. AI, by optimizing resource allocation, can elevate women’s status and expand their development. “The warmth of future technology is the warmth of women.”

Pushing through the “fourth wall” of the life stage

The *BRICS Women’s Development Report 2023* shows China’s digital economy accounts for nearly 40% of GDP (around 20% in many other countries). According to Alibaba Research, by March 2022, digital trade, e-commerce, and livestreaming created 57 million jobs for women; flexible work is on the rise, with roles like community commerce leads and content creators drawing more women.

By 2025, China’s digital economy is projected to employ about 120 million women. Consider Wang Zhuanlan, whose monthly income reportedly grew sixty-fold over sixteen years. Born in 1991 in mountainous Longnan, Gansu, she left school after sixth grade because her father thought girls’ education was useless. At 14 she moved north to work—starting as a nanny with just 280 yuan net her first month. She cycled through cleaning, waitressing, and sales. In 2015 she discovered an interest in UI design, enrolled in a six-month program, later earned a junior college credential through adult education, and in 2020 returned to rural Zhoukou, Henan, to learn short-video production. Her first home-renovation video on Douyin in 2021 drew 66,000 likes; followers watched a dilapidated house transform into a dream courtyard. She now has 400,000+ followers across platforms. For Wang, the internet has been a scaffold, toolbox, and stage of possibilities.

There are many rural women like her. Notably, Shen Dan—dubbed Fujian’s “Liziqi”—went from bankrupt single mother to founder of a cultural media company with 40 million followers across platforms, turning Wuyi Mountain aesthetics into a digital legend.

Professor Shi Ce of Northeast Normal University likens the internet to pushing open the “fourth wall” of women’s life stages—broadening horizons, enabling expression, and scaling empathy on a boundless, even global, stage. As digitalization and new business models grow, internet firms are creating jobs and flexible work that better balance family and career—helping women create value and realize dreams. *The Economist* once observed: “The next engine of growth will be driven by women,” underscoring women’s central role in the economy.

Douyin’s *2021 Women Data Report* shows 13.2 million women earned income directly from the platform in the prior year. Creators such as “Shu Zhong Taozi-jie” (Sichuan) and Qiao Xue (a Ningxia intangible-heritage leather artisan) launched new careers via short video. Kuaishou reports that women are about 60% of users; over 12 million women earn income or jobs on the platform. In short video, livestreaming, and social commerce, women’s employment exceeds men’s. On Xiaohongshu (RED), women are over 70% of users, and nearly 80% are post-90s—spawning fashion and lifestyle creators who turn content into entrepreneurship.

Importantly, the internet acts as an advantage amplifier for women in less developed regions. For example, national “March 8th Red Flag Bearer” Xia Hua (Eve Group) built a global designer open platform and databases for Miao embroidery, digitizing thousands of motifs, linking over 1,200 craft studios and 1,600 designers, and training 20,000 embroiderers—allowing women to earn income at home while preserving heritage. Thus, the internet promotes both women’s high-quality employment and the creative transformation of traditional culture.

At the 2023 BRICS Women’s Leadership Forum, Wang Shutong (Founder/Chair, DHGate) noted that women’s entrepreneurial opportunities have grown dramatically in the digital era—doubling during the pandemic in some cases—with women accounting for 40% of entrepreneurs or employees on DHGate platforms. “The internet gives women more flexibility and faces less visible bias—because the tools level the playing field,” she said.

A new cohort of women leaders in “Big Tech”

Women’s “traits” align with the internet’s emotional design, experience economy, and decentralized features. From once being relegated—as Mitchell polemically put it in *Iconology*—to “less-than-fully-human,” women have become mainstream actors in the internet era, reshaping destinies, mindsets, and, ultimately, the world.

According to 2023 ESG reports: women comprise 49.1% of Alibaba’s workforce and 41.9% of managers; Lenovo’s workforce is 37% women, with 21% women executives and ~30% women in technical roles; Baidu’s workforce is 39.3% women, with 43.9% women managers; Kuaishou’s workforce is 43.8% women.

A cohort of women executives is rising. In May 2023, JD.com appointed CFO Xu Ran as CEO—the first woman CEO in JD’s history and currently the most powerful woman among China’s internet majors. A dual B.S. in science and economics from Peking University and a PRC/US CPA, Xu spent nearly two decades at PwC before joining JD in 2018, becoming CFO in 2020 and leading major M&A, restructurings, and listings.

Alibaba, too, has had a woman at the helm: Dai Shan—one of the “18 founders”—worked her way from customer service to CEO of Taotian Group, which contributes nearly 70% of Alibaba’s revenue. Pinduoduo’s behind-the-scenes strategist Gu Pinging (Abu), ByteDance’s Zhang Nan (first female CEO of Douyin, later CEO of ByteDance China), Douyin E-commerce President Wei Wenwen, and Bilibili’s Vice Chair/COO Li Ni exemplify the ascent of women into the power centers of China’s internet economy.

As investor Aileen Lee once quipped, “Women are the routers and amplifiers of social networks—and rocket fuel for e-commerce.” In production and infrastructure, too, women increasingly supply “rocket fuel.” CASS researcher Jiang Qiping has argued that the internet’s deep linkage to feminism goes far beyond “women going online”: it signifies a shift in modern thought itself. On short-video platforms, many women creators promote safety awareness, independence, and resistance to beauty anxiety—e.g., “Ms. Silinglemon” (4.52M followers) showcases women’s realities and calls for courage and autonomy—leveraging the internet to challenge bias and expand rights, while building their own influence and capability.

Women-focused digital-public-benefit projects are also flourishing. Dong Kui, Secretary-General of the China Women’s Development Foundation, notes that initiatives like the “Digital Mulan” Women’s Development Conference showcase digital philanthropy, share experience, and mobilize goodwill—energizing the digital economy and helping build “Digital China.”

From an institutional perspective, provincial and municipal Internet Industry Women’s Federations have been established nationwide, strengthening guidance, services, and connections for women in the sector—creating opportunities and platforms so the federations become trusted “homes” for internet women.

Chinese women’s digital potential is boundless. In the next decade, women and the internet will write new legends—together.

Article URL (optional): <https://mp.weixin.qq.com/s/t-LXKvtahozMMTo0Ra56rw>

---

Article 18

Public Account Name: Gender studies horizon

Article Title: 2023 Forum on women pioneers in science and technology innovation

Date of Publication: 2023 December 15

Full Article Text:

On December 14, the 2023 Women Tech Innovation Pioneers Forum was successfully held at Zhongguancun Dongsheng International Science Park in Beijing. The forum was co-hosted by the Beijing Academy of Science and Technology, the Beijing Women’s Federation, the Beijing Association for Science and Technology, and the ZGC Global High-end Think Tank Alliance. As the launch event for the APEC PPSTI Fund Project—“Women’s Participation in STEM: APEC Dialogue on Practices that Promote Innovative Development”, the forum adopted the theme “Technology for Good · Humanity at Its Best.” It brought together over 100 guests, including three academicians—Qiao Jie, Duan Huiling, and Gong Qiaoyu—and 30 experts.

Through keynote speeches, roundtables, the conferral of “Women Tech Innovation Pioneer” recognitions, and a women-in-tech salon, the forum aimed to galvanize pioneering forces for women’s participation in STI, advance policy exchange and practice, and lay a solid foundation for implementing the APEC fund project. At the forum, Li Junkai—Project Lead, Secretary-General of the ZGC Global High-end Think Tank Alliance, and Director (Research Fellow) of the International and Regional Cooperation Center at BAST—presented the project report, *“Women’s Participation in STEM: APEC Dialogue on Practices that Promote Innovative Development.”*

Li noted that over the past two decades, women’s participation in STEM across APEC has remained low. According to the *APEC Women and the Economy Dashboard 2023*, only two APEC economies report a female participation rate in STEM reaching 50%. Most economies’ data collection is relatively delayed and focuses on four indicators: the share of female graduates in higher education, women’s employment in engineering/manufacturing/construction, the proportion of female researchers, and the proportion of women in R&D.

Li summarized policy measures from selected APEC economies in three areas: 1) Unlocking STEM potential through education. Mexico – Women in STEM, Future Leaders: Targets high-school girls via remote learning; establishes national training hubs and a digital education network to widen exposure to STEM. Each student is assigned a mentor—professional women, female PhD students, or professors—who provide one-on-one academic/career guidance and engage students through online/offline seminars to build interest and confidence. New Zealand – Nanogirl Labs: A social enterprise offering practice-based STEM professional development for primary and secondary teachers to build their confidence in supporting girls in STEM. In partnership with schools, it delivers hands-on STEM programs to at least 10,000 primary students annually; many modules require no prior STEM teaching experience, expanding reach by improving teaching tools and integration into primary curricula.

Secondly, supporting women’s STEM career development. New Zealand – Online “STEM Directory” (Ministry for Women): A searchable tool for girls and women to find STEM internships, jobs, programs, and policy initiatives, offering guidance for STEM learning and progression. Mexico – “Codigox”: Selects senior women with 10+ years in STEM from industry, academia, government, and civil society to mentor women professionals and young entrants one-to-one—bridging experience gaps and sharing sectoral resources. United States – Girls Who Code (GWC): Works to close the gender gap through programs in coding, web design, and robotics for middle and high school girls. It also funds professional-development and gender-inclusion training for tech companies, helping women upskill in the workplace.

Thirldy, raising women’s visibility and presence in STEM. Li highlighted that culture-building is pivotal: celebrating female role models, normalizing women’s STEM careers, and strengthening social foundations for participation. Australia: Across government departments, multiple initiatives support women in STEM. The Digital Transformation Agency runs the Women in IT Executive Mentoring and WITEM (Women in Technology and Management) programs to boost gender diversity among senior IT staff and enhance confidence and career progression. Singapore – POWERS (Promotion of Women in Engineering, Research, and Science): A joint program of the Ministry of Education and Nanyang Technological University to expand diversity in women’s STEM development. By building community, it provides a one-stop ecosystem from pre-university to post-doc, fosters positive learning environments, cultivates leadership among young women, and empowers them as agents of change. United States – NSF ADVANCE Partnership Project: Aims to increase the participation of women with disabilities in STEM academia and employment through communities of practice, dedicated funding for dissemination and implementation, and advisory/help desks connecting women to practical opportunities.

Li also underscored ongoing challenges to women’s advancement in STEM across APEC, as summarized in APEC’s “Women in STEM” studies. Environmental constraints include (but are not limited to): weak enforcement of legal gender-equality provisions; persistently low numbers of women researchers and officials; entrenched gender stereotypes; limited self-efficacy and confidence among women due to deep-rooted sociocultural norms; insufficient gender mainstreaming in STEM policies; complex multi-stakeholder cooperation mechanisms that are hard to implement; and the lack of basic livelihood and social security for women in poverty, which hinders their STEM development.

Article URL (optional): <https://mp.weixin.qq.com/s/b8uuq8W_Un5h2onVIKg90g>

---

Article 19

Public Account Name: Gender and society studies

Article Title: Why do highly educated women become full-time mothers?

Date of Publication:  2023 August 21

Full Article Text:

Shani Orgad, a scholar of gender and media at the London School of Economics, and a mother herself, observes the stark contrast between “working moms” and “non-working moms” during her daily school runs. The working mothers are dressed formally, rushing off to their jobs after drop-off, while the non-working mothers appear more relaxed, often in sportswear or casual jeans and T-shirts, ready for a workout, coffee, or brunch.

Orgad turned her analytical lens to these full-time mothers living in London’s affluent suburbs. Highly educated and once lawyers, doctors, accountants, teachers, or engineers, they had enjoyed the satisfaction and achievements of professional life. They had the competence to compete in the workplace and the economic means to outsource domestic chores and childcare. Why then, after marriage and childbirth, did they relinquish their careers and embrace the role of full-time homemakers? Were they simply privileged women, secure in their wealth, choosing to enjoy domestic life? Should they be seen as betraying the pursuit of gender equality and thus as targets of critique?

Why do highly educated women leave the workforce?

There are familiar explanations: a preference for family-centered life; a supposed maternal “instinct” or “natural” inclination; a decline in self-confidence and career investment after motherhood.

Orgad critiques such accounts that frame withdrawal from work as an entirely individual and autonomous choice. Drawing on C. Wright Mills’s notion of the *sociological imagination*, she argues for connecting what appear to be private troubles with broader social structures, and for locating the macro-social forces shaping personal predicaments. Her research investigates both lived experiences and cultural representations: through interviews capturing women’s self-expressions, and through analysis of policies, media narratives, and popular texts. She asks: *What kinds of experiences do these women have? How do they narrate their decisions and their domestic lives? How are women, work, and family portrayed in culture and policy, and how do such representations shape women’s choices?*

Behind “voluntary resignation”

The idealized image of women in Western media has shifted over time. In the 1950s–60s, the celebrated figure was the cheerful homemaker devoted to family. By the 1980s–90s, it became the “supermom,” juggling career and family with apparent ease. The “ideal woman” was imagined as overcoming obstacles to balance both spheres. Entering the 21st century, critiques emerged of such unrealistic ideals, emphasizing instead the complexity and diversity of women’s actual experiences and the constraints they face. In this climate, the traditional homemaker image was seen as outdated, and both culture and policy encouraged women’s workforce participation. So how did highly educated women with promising careers explain their decision to go home?

Orgad found that while these women had once pursued careers with confidence—sometimes even outpacing their husbands—the transition to motherhood exposed the incompatibility of work and caregiving. Ideal employees are expected to be constantly available and wholly dedicated. But as mothers, women frequently had to step back for childcare and education responsibilities, making overtime, meetings, and networking impossible. Their departure from work was thus not due to a preference for home life or a lack of ambition, but rather to workplaces and institutional arrangements fundamentally hostile to family life—for both themselves and their husbands.

Yet, in narrating their stories, the women often personalized the issue, blaming themselves: “I’m just not the type for being a working mom. I’m a perfectionist, I don’t have the ambition needed.” Against cultural depictions of the resilient, thriving working mother, they experienced exhaustion and guilt when unable to “balance it all.” External barriers were reframed as internal flaws, producing deep self-reproach.

Family CEOs, intensive mothering, and the hidden “wife role”

Orgad highlights the contradictory cultural messages about full-time mothers. On one hand, they are praised as “sweet mommies,” valued for nurturing and educating their children. On the other, abandoning years of education and career is criticized as deviant or wasteful. How did the women themselves navigate this ambivalence?

The interviewees feared being dismissed as idle or parasitic. As one put it: *“To just say ‘I’m a full-time mom’—it has no dignity, no value… worthless. People think you contribute nothing, you waste your degree, you live off others.”* To counter this stigma, many reframed themselves as “family CEOs,” running the household like a small enterprise. They redeployed professional skills in managing their “new job” and claimed legitimacy and dignity through this role. Distancing themselves from the image of the weary housewife, they stressed their focus on children’s education, school activities, and extracurricular coordination, aligning with the contemporary ideal of intensive mothering.

“Doing it for the children” dominated their accounts. Motherhood was the primary identity after leaving work. Yet Orgad perceptively notes that beneath the maternal narrative lies the story of the wife. Their withdrawal was also about supporting husbands’ demanding careers. But unlike the “good mother,” the “dutiful wife” identity was fraught with ambivalence—signifying regression to the traditional “male breadwinner, female homemaker” model and unequal gendered divisions of labor. Motherhood was celebrated, but wifely devotion was stigmatized. As Orgad puts it: *“The wife remains an unwelcome, low-visibility identity: in the 21st century, women’s primary roles are worker and mother, not wife.”* Thus, the “wife role” was repressed, veiled by maternal discourse.

Looking ahead: is “mompreneurship” the solution?

Asked about the future, most mothers expressed a wish to return to paid work, to regain the recognition and self-worth it once provided. Their envisioned pathway mirrored media and policy prescriptions: mom entrepreneurship—entering the gig economy, blending home and public work, combining economic activity with caregiving. The media portrays this as an ideal picture: working from home, flexibly managing both tasks and children.

Yet Orgad critiques this vision as a fragile illusion. It glosses over the stressful, chaotic realities of caregiving and the precarious conditions of gig work—overwork, lack of protection, high failure rates. Crucially, it fails to challenge the assumption that mothers remain the primary, if not sole, caregivers. *“It is an individual-level fantasy solution to structural problems.”*

Her research reveals that highly educated women’s retreat to the home is far from a purely free choice. Structural constraints play decisive roles. These women were privileged—well-educated, supported by husbands’ high incomes. Yet if even they cannot resist systemic inequities or articulate their aspirations, it is even harder for less advantaged women.

In closing, Orgad returns to Mills’s sociological imagination: once we recognize that personal troubles are shaped by broader social forces, solutions must be collective, not individualized. She calls for action to link workplace equality with domestic equality, to resist long-hours, high-demand work cultures, to challenge the devaluation of care work, and to reject the notion of mothers as the default or sole caregivers.

Article URL (optional): <https://mp.weixin.qq.com/s/5Sy6p3nYhC4ZLy4DwNgBag>

---

Article 20

Public Account Name: Gender and society studies

Article Title: Constructing the new mother: The formation and practice of child-rearing knowledge in modern China (1900–1937)

Date of Publication:  2023 October 27

Full Article Text:

Teaching New Knowledge of Childbearing

*"Water my good seedlings, harvest your fine fruits; but those of foreign seed, weed them out."*

This poem, titled *“Lament for the Female Race” (Ai nüzhong)*, was published in *Women’s World* (1903) by Ding Chuwuo. From its opening lines, the essay sounded an alarm: under the struggle of “foreign races,” the weaker are the first to be defeated, and among the weak, women are the weakest and thus the earliest to fall. According to Ding, China was facing a crisis: *“We have no time to lament for the male race, we must first lament for the female race.”* Why first lament women? Because, he argued, one of the root illnesses of China’s national mothers lay in their ignorance of how to nurture the weak species. He pointed out that Westerners had abundant childrearing books, discussing heredity, the behavior of pregnant women, swaddling infants, children’s nature, and every detail of diet, hygiene, and daily care. By contrast, Chinese mothers were unaware of their natural “maternal power” of nurture, unable to exert any force in “guiding the evolution of the species.” As a result, the Chinese people appeared bent-backed, sickly, and feeble, while white people were described as strong, upright, vigorous, and swift.

This type of discourse—linking women’s maternal role to “strengthening the nation and preserving the race”—was a core motif of late Qing nationalist rhetoric. In this national movement, women were charged with the task of *baozhong* (race preservation), acting as “mothers of the nation,” with the ultimate goal of “giving birth to strong sons.” Reformers earlier on had already adopted “strengthen the nation and preserve the race” as a slogan.

One prominent example was Kang Youwei’s *Book of Great Unity* (*Datong shu*), which designed a comprehensive public childcare system beginning from the moment of conception. He envisioned a “Human Foundation Institute” (*Renben yuan*) where pregnant women would receive instruction and nurture from the earliest stages of pregnancy. This plan drew from ancient Chinese ideas of *taijiao* (prenatal education), which viewed the fetus as the foundation of human life and moral cultivation, while also incorporating Western notions of racial improvement and eugenics.

In Kang’s design, the *Renben yuan* would provide pregnant women with spacious, clean, well-ventilated living quarters, gardens for viewing, and access to art and music to cultivate their temperament. Specialized personnel were assigned: *female physicians* to monitor maternal and fetal health twice daily; *female teachers* to instruct in moral tales and refined knowledge; *female nurses* to provide lessons in hygiene, fetal care, childbirth, and childcare; and *female attendants* to supervise and arrange daily routines. Whether within the institute or in the broader society, pregnant women were to be treated with respect, because “a pregnant woman carries Heaven’s mandate to give life—she is a public mother of the people.”

Although Kang’s blueprint was highly idealized and far from reality, it reflected a fusion of Chinese and Western knowledge: combining the traditional emphasis on *taijiao* as the indispensable first stage of education with modern Western “methods of racial improvement.”

New Reproductive Knowledge and Women’s Journals

If the preservation of the race required vigilance, then education had to begin from the moment of pregnancy. How to nurture the fetus, what dietary and daily habits to follow, how to ensure safe childbirth, what remedies for difficult labor or excessive fertility—these concerns were not absent from traditional China. Yet, from the late Qing onward, under the influx of Western knowledge, reform-minded intellectuals actively imported reproductive science and disseminated it to women through magazines.

This chapter, using women’s journals as primary sources, examines the features of this new knowledge: Prenatal education of the mind – exploring both negative and positive approaches. Nurturing of the body – addressing maternal diet, lifestyle, and physiology. Changes in birthing practices – focusing on preparation for labor, the act of delivery, and postpartum care. New debates on fertility control – including advocacy of birth limitation, attitudes toward abortion, and attribution of infertility.

Genetics and Eugenics: A New Understanding

Teaching women about heredity and eugenics was a novelty of the modern era, absent in traditional learning. By the early 20th century, “eugenics to save the nation” (*you sheng jiu guo*) had become both a slogan and a criterion for spouse selection.

In 1916, Chen Qi published “On Prenatal Education” in *Women’s Magazine*. He compared traditional Chinese *taijiao* with Western theories of heredity. While Chinese prenatal education emphasized moral cultivation—expecting pregnant women to remain calm, avoid evil sights and sounds, and embody virtue—Western “prenatal education” encompassed three theories: Eugenics (liangyu shuo): focusing on physical fitness and the selection of robust mates. Heredity: asserting that parental illness predisposed children to weakness (e.g., tuberculosis). Chen cited Western examples such as Napoleon’s vigor, attributed to his parents’ wartime activity before his birth, and the assassin of U.S. President McKinley, whose mother had once contemplated abortion—supposedly “transmitting” homicidal impulses to the fetus. Love and health theory: advocated by Ellen Key, stressing that ideal children required parents who married for genuine love and were both physically healthy.

Although Chen regarded Western theories as overly biased toward physical strength, he still argued that China, branded the “Sick Man of East Asia,” could only be revitalized by strengthening physical fitness.

From today’s perspective, some of these examples—such as the idea that a mother’s fleeting thoughts of abortion could be inherited by her child—seem absurd. Yet they reveal how imported ideas, often uncritically absorbed, circulated in print as scientific truth.

In 1931, Y.D. published “Eugenics and American Marriage Law” in *Women’s Magazine*, systematically introducing the origins, methods, and legal frameworks of eugenics, including U.S. state-level prohibitions on “inferior” marriages and the criticisms they drew. In the same year, Zhu Wenyin’s “Prenatal Education and Eugenics” argued that traditional Chinese *taijiao* was insufficient, and that only by combining eugenics and genetics could humanity be fundamentally improved, producing a superior “child of man.”

Women as Biological Mothers of the Nation

Disciplines like genetics and eugenics, absent in traditional China, were now disseminated through women’s magazines, teaching mothers to understand their own bodies and their biological role in producing the next generation. The health and strength of mothers were now directly tied to national competitiveness.

As debates on heredity and eugenics swept the globe, women worldwide—Chinese women included—were drawn into a racial survival struggle. Their biological role as mothers, framed as the guardians of the nation’s future, was thereby further intensified and naturalized.

Article URL (optional): <https://mp.weixin.qq.com/s/LZEsXg76n4vby6sDBzNq6A>

Article 21

Public Account Name: Gender and society studies

Article Title: “Leftover women” in matchmaking corners: caught between tradition and modernity

Date of Publication: 2023 September 12

Full Article Text:

Some time ago, a skin-care brand released a viral ad titled “She Finally Went to the Matchmaking Corner.” In Shanghai’s People’s Park, parents gather with their children’s bios and photos to arrange matches. Under mounting family pressure, a group of “leftover women” decide to go there themselves and write down their own terms. Closely related to that ad is the short piece we introduce today—Qian Yue, PhD, translates and summarizes an article by Ji Yingchun published in the *Journal of Marriage and Family*, “Leftover” Women in Shanghai: Navigating Tradition and Modernity.

First, clarifying terms: what is “traditional” and what is “modern”? Broadly, “traditional” refers to arranged marriages, parental intervention, male dominance, and multigenerational households. “Modern” points to love-based marriage, individual freedom, women’s independence and self-development, and nuclear families. These are often cast as two poles on a linear path from tradition to modernity. Yet growing research shows that in changing marital/familial values and behaviors, elements of “tradition” and “modernity” frequently coexist—even within the same person.

The study’s subjects are the very “leftover women” featured in the ad. The researcher conducted one-on-one interviews in Shanghai with 30 never-married women: one-third Shanghai natives; ages 26–40 (most in their early 30s); nearly all college-educated, with half holding master’s or doctoral degrees; professionally successful (physicians, engineers, university faculty, etc.).

What common threads run through their lives? Firstly, parental pressure. All reported parental pressure to marry, yet insisted they would “wait for the right person.” “My mother is frantic about my marriage. She scolds me, says I’ve disappointed her, lost her face, and made her unhappy. She introduces any single man she can find, regardless of fit. I feel awful imagining I’ve let down the people I love.”

Do they want to marry? Most said yes. “I have a family—my parents—so I can’t only pursue my own ideal life. My mom thinks I must marry now, no matter who. Our goal is the same—marriage—but our ideas are totally different. I do want to marry; I just haven’t found the man I’m willing to marry.” Thus, highly educated, modern women still consider family central. While arranged marriages are now rare, traditional parental expectations remain powerful. These women share their parents’ end goal (marriage) yet hold to more idealized, love-centered views—embodying a tension between tradition and modernity.

Secondly, a double standard of aging. Interviewees observed that women face harsher age penalties than men. “As you get older without finding someone, women panic—men don’t. When I was in my 20s, many tried to set me up; now, almost no one. Men at 40 can still date widely. A colleague divorced two years ago and has remarried—he’s nearly 50, his wife was born in 1985. I’ve never seen the reverse.” The enduring ideal of “talented man, beautiful woman” privileges men’s time-dependent “merit/money” and women’s youth/looks. Without a shift among men, women alone can’t overturn this norm.

Thirdly, pushed to “marry up”. Career-successful women reject male-superior gender hierarchies and are open to marrying men who earn less. Yet they worry such men may feel insecure, straining the relationship. Many also noted that men often reject older, equally aged, or more successful women. If men cling to traditional gender and matching norms, independent, equality-oriented women are more likely to remain single.

Fourth, “matching doors” (men dang hu dui), redefined. Interviewees increasingly value compatibility, but in a modernized sense. “Matching doors” now means shared values, goals, and communicative culture, often facilitated by similar family backgrounds. One woman broke up after her Shanghai boyfriend’s parents looked down on her non-Shanghai family: if his family does not respect her parents, she will not marry him. For the only-child generation, family influence remains strong; they link modern ideals of egalitarian intimacy with a reframed notion of social parity.

Fifth, balancing family and career. Many take pride in careers and achievement, yet aspire to be “good wives and mothers,” striving for balance: “Men and women play different roles. Even if I’m a strong, successful woman, I still like men with drive. As a wife, I’ll help balance his work and family—I’ll prioritize home.” Thus, even as they succeed publicly, their expectations for the private sphere often remain traditional.

Sixth, clashing gender views. Women felt many men retain traditional attitudes and do not appreciate women’s success: “People say, ‘Act more feminine. If you’re too assertive, you’ll never find a boyfriend.’ Other women admire my English, education, and job. Men tell me they worry they can’t ‘control’ me—they want someone who won’t surpass them. Why control? Why not appreciate me as an equal?”

These single, well-educated, urban professional women live at the sharp intersection of tradition and modernity: They face parental pressure yet insist on individualized, love-based ideals. They confront bias around women’s education, careers, and age, yet reject marrying only older/more “successful” men. They redefine “matching” as value and cultural compatibility, not merely wealth or status. They are independent and successful publicly, yet often maintain traditional private-sphere expectations. They pursue gender equality more strongly than men and criticize men’s conservative norms. The researcher argues that China’s so-called “leftover women” are innovative agents: facing resurgent patriarchal expectations, they actively and strategically craft lives that weave together the modern and the traditional.

Article URL (optional): <https://mp.weixin.qq.com/s/xJ4ru-Yb4pHOHdKMAj8rUQ>

Article 22

Public Account Name: Gender and society studies

Article Title: Revealing hidden labor

Date of Publication: 2023August 14

Full Article Text:

Why Household Labor Became a Scholarly Topic

When you think of “housework,” you might picture laundry detergent ads: a smiling mother effortlessly removes stains, proud of the fresh laundry. Or perhaps you think of it as women’s “natural” talent—an act of love, willingly given. Or you might dismiss it: “just a housewife,” “just chores.”

Yet household labor is repeated every single day, sustaining daily life and shaping everyone’s routines. Can this seemingly trivial activity be elevated into an academic research subject? For a long time, sociology ignored it—within a male-dominated discipline, women’s experiences were marginalized. In the 1970s, British sociologist Ann Oakley broke new ground. She carried out an exploratory, methodologically innovative study: listening to housewives’ own accounts of domestic labor, and analyzing it as *work*—just like any other kind of work in modern society.

Oakley interviewed 40 London housewives from working- and middle-class backgrounds, aged 20–30, all married with at least one child under five. She argued that even such a small-scale study advanced our understanding of women’s lives and urban family life, and contributed to sociological knowledge. Her research described housewives’ lived experiences and attitudes toward housework, and analyzed how factors like class, education, household division of labor, and patterns of social interaction shaped their perspectives—explaining why housewives’ attitudes and practices varied.

Viewing Housework as Work

People often use “housework” as a catch-all for everything women do to keep households running. Oakley showed it is not a monolith but a set of distinct tasks: cleaning, shopping, cooking, dishwashing, laundry, ironing. Housewives expressed different feelings about each. Positive aspects included: social interaction, maintaining good mood, enough time, supportive environments or tools, sufficient money, and appreciation from others. Negative aspects included: monotony, poor conditions or inefficient tools, bad moods, children interrupting, lack of time, social isolation, and juggling with paid work.

Oakley observed that women described housework much like they described other jobs—they saw it as *work*. Housewives were not “the idle class” of stereotypes. Another stereotype cast housewives as *oppressed workers*, enslaved in degrading, self-negating toil. Oakley drew on the concept of job satisfaction from sociology of factory and paid labor, applying it innovatively to evaluate “housework satisfaction.” This gave women the chance to voice real feelings. If housework were simply women’s “duty” as wives and mothers, they should be content—yet they weren’t. As one respondent put it:

“When you know you’re supposed to feel satisfied, what’s the point of admitting you aren’t?” Contrary to claims that discontent was unique to educated middle-class women, Oakley found dissatisfaction widespread across classes. Housewives contrasted their paid work (past or present) with unpaid domestic labor, which lacked pay, recognition, and social value. Both working-class women in repetitive jobs and middle-class women in higher-status professions expressed similar frustrations.

Work usually implies external norms—when, how, and to what standards tasks must be done. Housework, by contrast, seemed unregulated. Housewives often said they were their “own boss.” But Oakley found that women set their own standards and routines, which became binding. This revealed that housework, too, has a structured logic, with implicit rules, much like other work.

She highlighted two paradoxes of “autonomy”: Standards and routines appear self-chosen but become objectified obligations, binding the women themselves. This process undermines autonomy—they became their own bosses, judges, and reward-givers, bound by self-imposed discipline.

Housework Bound to “Femininity”

Preparation for housework began in childhood socialization. Oakley examined how girls’ identities and “femininity” were tied to family life and housework. Interviewees recalled early chores, measuring themselves against their mothers—imitating or resisting her model. Household duties were gendered: seen as “feminine,” integral to identity. Early acceptance of housework shaped later behavior, regardless of class background.

In marriage, how is housework divided? Does women’s paid employment lead to equality? Do men take more responsibility? Oakley’s data showed men’s participation remained rare. Equality in leisure or decision-making did not predict equality in housework or childcare. Traditional inequalities persisted. Gender role norms powerfully shaped behavior: women were still seen as naturally suited to home and children. Even as men helped more, underlying assumptions about gender roles remained unchanged. Women still bore primary responsibility. This bias existed not only among families but also in sociology’s own research frameworks.

Contemporary Studies of Housework

Women’s domestic labor has long been hidden under assumptions of wifehood and motherhood. Oakley made it visible, showing that women’s family responsibilities remain a barrier to gender equality. Combating discrimination requires theory, policy reform, cultural change, and—equally—greater self-awareness among women of how femininity is bound to domesticity.

Today, housework is recognized as a major research theme in gender equality, family, and marriage studies. It features in large-scale surveys, analyzed with advanced statistics, documenting time use, division of labor, men’s participation, and effects on labor market outcomes. With aging populations, smaller families, and childcare/eldercare shortages, outsourcing and marketization of domestic labor have become new topics: when housework becomes paid work, how does it affect family relations, laborers’ experiences, and the home-service industry? Scholars call for public policy measures to support families and advance gender equality.

Article URL (optional): <https://mp.weixin.qq.com/s/fx_8F0VATQnmEuk_YzILew>
